# Integrated lipids biomarker of the prediabetes and type 2 diabetes mellitus Chinese patients

**DOI:** 10.3389/fendo.2022.1065665

**Published:** 2023-01-20

**Authors:** Jiaying Yang, Mei Wang, Dawei Yang, Han Yan, Zhigang Wang, Dan Yan, Na Guo

**Affiliations:** ^1^ Experimental Research Center, China Academy of Chinese Medical Sciences, Beijing, China; ^2^ College of Pharmacy, Heilongjiang University of Traditional Chinese Medicine, Heilongjiang, China; ^3^ LU-European Center for Chinese Medicine and Natural Compounds, Institute of Biology, Leiden University, Leiden, Netherlands; ^4^ Zhong Yuan Academy of Biological Medicine, Liaocheng People’s Hospital, Liaocheng, Shandong, China; ^5^ Beijing Institute of Clinical Pharmacy, Beijing Friendship Hospital, Capital Medical University, Beijing, China

**Keywords:** prediabetes, type 2 diabetes mellitus, lipidomics, ceramide, UHPLC-MS

## Abstract

**Introduction:**

Dyslipidemia is a hallmark of T2DM, and as such, analyses of lipid metabolic profiles in affected patients have the potential to permit the development of an integrated lipid metabolite-based biomarker model that can facilitate early patient diagnosis and treatment.

**Methods:**

Untargeted and targeted lipidomics approaches were used to analyze serum samples from newly diagnosed 93 Chinese participants in discovery cohort and 440 in validation cohort *via* UHPLC-MS and UHPLC-MS/MS first. The acid sphingomyelinase protein expression was analyzed by Western blot.

**Results and Discussion:**

Through these analyses, we developed a novel integrated biomarker signature composed of LPC 22:6, PC(16:0/20:4), PE(22:6/16:0), Cer(d18:1/24:0)/SM(d18:1/19:0), Cer(d18:1/24:0)/SM(d18:0/16:0), TG(18:1/18:2/18:2), TG(16:0/16:0/20:3), and TG(18:0/16:0/18:2). The area under the curve (AUC) values for this integrated biomarker signature for prediabetes and T2DM patients were 0.841 (cutoff: 0.565) and 0.894 (cutoff: 0.633), respectively. Furthermore, theresults of western blot analysis of frozen adipose tissue from 3 week (prediabetes) and 12 week (T2DM) Goto–Kakizaki (GK) rats also confirmed that acid sphingomyelinase is responsible for significant disruptions in ceramide and sphingomyelin homeostasis. Network analyses of the biomarkers associated with this biosignature suggested that the most profoundly affected lipid metabolism pathways in the context of diabetes include *de novo* ceramide synthesis, sphingomyelin metabolism, and additional pathways associated with phosphatidylcholine synthesis. Together, these results offer new biological insights regarding the role of serum lipids in the context of insidious T2DM development, and may offer new avenues for future diagnostic and/or therapeutic research.

## 1 Introduction

T2DM makes up over 90% of human diabetes cases (1), and is among the most rapidly growing threats to human health throughout the globe (2). T2DM develops over several years in prediabetic individuals (3, 4), early diagnosis and treatment can effectively prevent the development of diabetes. Therefore, the detection of reliable biomarkers associated with prediabetes and T2DM is an area of active research, and multiple biomarkers including fasting blood glucose (FBG) and glycated hemoglobin A1c(HbA1c) (5–8) have been proposed as tools to assess the risk of diabetes (3–8). While valuable, however, these biomarkers fail to fully capture the complexity of T2DM development, and may also fail to detect at-risk individuals prior to disease onset (4, 9–11).

Dyslipidemia, and lipoprotein metabolism abnormalities are commonly detected in those with diabetes (12–14). Detecting these shifts in lipid profiles thus represents a promising approach to identifying high-risk patients at earlier time points. Lipidomic analyses of overall lipid profiles can also offer additional insight into the pathophysiology of diseases (15–17), including diabetes (15, 18–22). Several lipidomics studies have provided evidence that comprehensive lipid profiles have the potential to improve diabetes risk assessment relative to the use of conventional clinic indices alone Certain subclasses of lipids including ceramides, sphingolipids, phospholipids, triglycerides (TGs) having been linked to human prediabetes and T2DM (23–34)s T2DM is highly prevalent in European nations (35–37), and the human serum lipidome is highly complex (38, 39), a majority of recent studies have employed lipidomics approaches to analyze the serum lipid profiles of European individuals with prediabetes and T2DM (40–42). However, diabetes rates are rising rapidly in China such that it is now home to the highest global diabetes incidence (43), with prediabetes affecting a remarkable 35.7% of the population (44). Chinese dietary composition and obesity rates are very distinct from those in Western nations, and relative to European T2DM patients, those from China are often diagnosed at younger ages and with lower body mass index (BMI) values (45). As such, more in-depth analyses of the roles of endogenous lipids in the pathophysiology of prediabetes and T2DM in Chinese patients is essential to guide the development of novel preventative measures or treatment strategies. Furthermore, in recent years, an increasing number of studies have shown that synthesis of ceramide by sphingomyelinase hydrolysis sphingomyelin is considered to be one of the major causes for insulin resistance (29).

Sphingomyelinase-regulated balance of ceramides and sphingolipids plays an important role in many diseases (30, 46, 47). Sphingomyelinase especially acid sphingomyelinase has a central function for the re-organization of molecules within the cell upon stimulation and thereby for the response of cells to stress and the induction of cell death but also proliferation and differentiation (31). The role and mechanism of ASM research in many diseases has made great progress, which fully confirmed the important role of ASM/ceramides pathway in T2DM, However, there are few studies on prediabetes. It is important to further study the exact regulation mechanism of ASM pathway in pathophysiology of prediabetes. Previous studies have suggested that patients with long-standing T2DM and had worse metabolic profiles when compared with the newly diagnosed (48), and multiple complications such as chronic kidney disease (CKD) and diabetic kidney disease (DKD) remain common in diabetics in the decade after diagnosis (49). In addition, long-term use of hypoglycemic drugs such as metformin and acarbose also could alter the lipid profile of human (50, 51), revealing metabolic changes of diseases. Thus, it is very key for study of lipid metabolic profiles of participants with prediabetes and T2DM with the newly diagnosed. Untargeted lipidomics analyses are limited by their narrow linear range, poor reproducibility, and low sensitivity (52, 53), whereas targeted approaches exhibit reduced metabolomics coverage such that they have the potential to miss metabolites of interest. As such, combining targeted and untargeted lipidomics strategies can overcome potential misannotation owing to the structural diversity and complexity of lipid molecules, thereby enabling the better confirmation of results to offer insight into lipid metabolism in the pathophysiology of metabolic diseases.

Herein, we employed untargeted and targeted UHPLC-MS and UHPLC-MS/MS approaches to analyze the serum lipid profiles of Chinese individuals with newly diagnosed patients or without prediabetes or T2DM. Subsequently, western blot analysis of ASM in different ages of GK rats was performed in order to explore and confirm whether ASM is responsible for significant disruptions in ceramide and sphingomyelin homeostasis and the important role of ASM/ceramides pathway in prediabetes and T2DM patients. The resultant data were analyzed with both commercial and in-house software applications. The overall goals of this study were to systematically screen for potential lipid biomarkers associated with prediabetes and T2DM incidence in Chinese patients in order to both better understand lipid pathway dysregulation and to develop a new integrated biosignature that may aid in diagnosing these conditions.

## 2 Materials and methods

### 2.1 Participant recruitment and grouping

All study participants were recruited from Beijing Shijitan hospital at the Capital Medical University (Beijing, China), Beijing Jiao Tong University Community Health Center (Beijing, China), The First Affiliated Hospital of Zhengzhou University (Henan, China), The First Affiliated Hospital of Henan University of Chinese Medicine (Henan, China), and Kaifeng Hospital of Traditional Chinese Medicine (Henan, China). All subjects underwent a physical examination during which their height, weight, and BMI were recorded. They then completed a face-to-face interview during which they detailed their demographics, medical history, family medical history, and other lifestyle factors. Blood samples were additionally collected to measure participant plasma total cholesterol (TC), High density lipoprotein (HDL), Low density lipoprotein (LDL), triglyceride (TG), FBG, alanine transaminase (ALT), and aspartate transaminase (AST) levels. Individuals were eligible for final study enrollment if they met the following criteria: (1) patients exhibited an FBG > 7.0 mmol/L (54) or met the diagnostic criteria for prediabetes (FBG: 5.6-6.9 mmol/L) (55, 56); (2) patients were 20-70 years of age. Patients were excluded if they: (1) exhibited a history of cardiovascular or cerebrovascular events; (2) had impaired liver/kidney function; (3) had a fasting triglyceride level ≥ 10mmol/L; (4) suffered from other endocrine, autoimmune, renal, cancerous, or otherwise serious diseases; (5) were undergoing treatment with antibiotics, glucocorticoids, or traditional Chinese herbal medicines; (6) were pregnant or expecting to become pregnant; (7) were currently breastfeeding; (8) suffered from mental health conditions; (9) declined or were unable to comply with study dietary guidelines; or (10) suffered from severe infectious diseases. Based upon this criteria, participants were grouped into control (n=35), prediabetes (n=31), and T2DM (n=27) discovery cohorts as well as control (n=150), prediabetes (n=170), and T2DM (n=120) validation cohorts. The Ethics Committee of Scientific Research, Beijing Shijitan Hospital, Capital Medical University approved this study, and all participants provided written informed consent to participate.

### 2.2 Chemicals and materials

Liquid chromatography/mass spectrometry (LC/MS)-grade methanol, acetonitrile, 2-propanol, ammonium formate, and HPLC-grade methyl tert-butyl ether (MTBE) were obtained from Fisher Scientific (PA, USA). LC/MS-grade ammonium formate was from Sigma-Aldrich (MO, USA). A Milli-Q system (MA, USA) was used to prepare ultra-pure water (18.2 MΩ).

Lysophosphatidylcholine (LPC 19:0), Phosphatidylethanolamine (PE 12:0/13:0), Ceramide (Cer d18:1/17:0), Sphingomyelin (SM (d18:1/12:0), TG (15:0/15:0/15:0), and Phosphatidylcholine (PC 12:0/13:0) were purchased for use as internal standard (IS) compounds from Avanti Polar Lipids (AL, USA). Antibodies used in this study were rabbit anti-acid sphingomyelinase polyclonal antibody (Absin, Shanghai, China).

### 2.3 Sample preparation

For untargeted lipidomics analyses, serum (10 μL) and cold methanol containing IS compounds (125 μL) were mixed for 30 s, followed by the addition of MTBE (500 µL). Lipids were then extracted by constantly agitating these samples for 20 min at room temperature, followed by the addition of water (125 µL), shaking for 30 s, and centrifugation at 16,826×g for 10 min at 4°C. For untargeted analyses, 200 µL of the resultant supernatant was dried with a concentrator prior to resuspension in a 100 µL volume of water: isopropanol: acetonitrile (5:30:65 (v/v/v). These samples were then agitated for an additional 30 s, followed by centrifugation at 16,826×g for 5 min at 4°C. The isolated supernatants were then evaluated *via* ultra-performance liquid chromatography/time of flight-mass spectrometry (UHPLC/TOF-MS) as soon as they had been collected. For targeted lipidomic analyses, a 100 µL volume of the supernatant prepared above was dried, resuspended in a 200 µL volume, and analyzed *via* UHPLC/MS-MS. A quality control (QC) serum sample was also generated for further analyses by mixing together 5 μL of each serum sample. These QC samples were processed for analysis in the sample manner as individual samples throughout the duration of our analyses. The QC samples were injected every 10 injections, and analyzed 10 times (discovery cohort) and 44 times (validation cohort) between samples to verify the stability of the LC-MS system respectively.

### 2.4 Untargeted and targeted UHPLC-MS lipidomics analyses

An Acquity UPLC BEH C_8_ column (2.1 × 100 mm, 1.7 μm) was used for lipid separation using a mobile phase composed of 5 mM ammonium formate with acetonitrile/water (A, 6:4; v/v) and 5 mM ammonium formate with isopropanol/acetonitrile (B, 9:1; v/v). Linear elution gradient settings for separation were: 0–1.0 min, 100% A; 1.0–2.0 min, 100–70% A; 2.0–12.0 min, 70–30% A; 12.0–12.5 min, 30–5% A; 12.5–13.0 min, 5–0% A; 13.0–14.0 min, 0% A; 14.0–14.1 min, 0–100% A; and 14.1–16.0 min, 100% A. The column was maintained at 55°C. An ACQUITY UPLC connected to a XEVO-G2XS quadrupole time-of-flight (QTOF) mass spectrometer (Waters, Manchester, NH, USA) in ESI+ mode was used for untargeted lipidomics analyses with the following settings: desolvation gas at 800 L/h and 400°C; cone gas at 50 L/h; source temperature at 100°C; capillary and sampling voltages of 2,000 V and 40 V, respectively. Mass data were acquired in MS^E^ mode at a ramping collision energy of 10–60 V. Data accuracy was ensured using a LockSpray™ source, with the (M+H)+ ions of leucine-enkephalin being set at m/z 556.2771 for the lock mass in ESI+ mode. Sample profiling data were acquired from 50 - 1,200 Da. A UHPLC system (Waters Acquity) with a Xevo TQ-S mass spectrometer and an ESI ionization source was used for targeted lipidomics analyses conducted using multiple reaction monitoring (MRM) in positive ion modes.

### 2.5 Experimental animals and adipose tissue collection

Goto–Kakizaki (GK) rat is one of the best characterized animal models of spontaneous T2DM. This model was established by selectively breeding of normal Wistar rats with signs of impaired glucose tolerance (57). It displays hyperglycemia, impaired glucose tolerance, insulin resistance and also defects in insulin secretion. In most of the GK studies, Wistar rats of outbreed origin are used as control animals (58). In additition, GK pups become overtly hyperglycemic for the first time after 3–4weeks of age only (i.e., during the weaning period). The occurrence of basal hyperglycemia and diabetes in the GK rat is therefore preceded by a period of prediabetes (22-28 days) (59). This study involved 10 T2DM GK male rats (12 week); 10 prediabetic GK male rats (3 week) and 10 control Wistar male rats obtained from Nanjing Junke Biotechnology Co., Ltd. (Jiangsu, China). A GLU Assay Kit (KOFA, China) and an automatic biochemistry analyzer (Hitachi 7020, Tokyo, Japan) were used to measure glucose concentration of GK rats. Rats were anesthetized using pentobarbital sodium (3%, 0.2 ml/100 g) and sacrificed by abdominal aortic exsanguination. After the adipose tissues of the rats was collected, snap-frozen in liquid nitrogen, and transferred to a -80 freezer until analysis. The experiments were approved by the China Pharmaceutical University Animal Care and Use Committee.

### 2.6 Western blotting

The ASM protein expression was analyzed by Western blot. The adipose tissue were washed twice by phosphate-Buffered Saline (PBS) and lysed in radio immunoprecipitation assay (RIPA) lysis buffer. The protein concentrations were determined by the bicinchoninic acid (BCA) protein assay kit. 30ul proteins were separated by 10% sodium dodecyl sulfate-polyacrylamide gel electrophoresis (SDS-PAGE) and transferred to polyvinylidene fluoride (PVDF) membranes, and blocked by immersing the membrane completely in 5% bovine serum albumin-tris buffered saline tween (BSA-TBST) and incubating on a horizontal shaker for 1 h. The membranes were probed with the primary antibodies of ASM (1:1000), overnight at 4°C followed by incubation with the secondary antibody goat anti rabbit IgG (H+L) at room temperature for 1 h. glyceraldehyde-3-phosphate dehydrogenase (GADPH) were used as control protein. The resulting complexes were visualized using chemoluminescence Western blotting detection reagents enhanced chemiluminescence (ECL). The blot was detected by chemiluminescent detection systems with LumiGlo and Peroxide (1:1, BU). Densitometric analysis of the images was performed with Image Pro Plus software (v.6.0) (Media Cybernetics, Inc, MD, USA).

### 2.7 Statistical analyses

The Waters MarkerLynx software (Waters; Micromass MS Technologies, Manchester, UK) was utilized to analyze data from untargeted lipidomics analyses in an effort to identify serum biomarkers specifically associated with prediabetes and T2DM patients. Waters Progenesis QI Applications Manager (v2.3) was utilized for peak finding, filtering, and alignment with the following data collection parameters: retention time = 0.5-15.5 min; mass = 50-1,200 Da. SIMCA-P (v13.0) (Umetrics, Umea, Sweden) was used to conduct multivariate statistical analyses of the resultant data. Partial least squares discriminant analysis (PLS-DA) was conducted in order to visualize the global metabolic difference of individuals between the control, prediabetes and T2DM groups. To validate the PLS-DA model, permutation tests were performed (n = 200). The Skyline software (v21.1) (MacCoss Lab; WA, USA) was used for data acquisition and peak processing for targeted lipidomics analyses. MetaboAnalyst 5.0 Web service (www.MetaboAnalyst.ca) was used to normalize raw data for next statistical analyses. Data have a normal distributed by Kolmogorov-Smirnov test and Quantile-Quantile plots (Q-Q plots). Independent samples t-tests and ROC curve analyses were performed using SPSS (v26.0) (IBM, NY, USA) P-value < 0.05 corrected by FDR was used as the cutoff for significance of differential metabolites. Column diagrams and forest plots were drawn by GraphPad Prism 9.0 (GraphPad Software Inc., USA). Python was used to generate heat maps highlighting correlations between putative biomarkers and specific clinical parameters calculated based upon Pearson correlation coefficients.

## 3 Results

### 3.1 Patient characteristics

In total, 533 participants ultimately met the criteria for enrollment of this study, of whom 93 were included in a discovery cohort (control = 35, prediabetes = 31, and T2DM = 27) and 440 were included in a validation cohort (control = 150, prediabetes = 170, and T2DM = 120). Patient clinical characteristics are summarized in **Table 1**. As expected, patients in the prediabetes and T2DM groups in both cohorts exhibited higher FBG and TG concentrations relative to controls. T2DM patients also exhibited a significant reduction in HDL content relative to control participants (P = 0.01), with a similar downward trend being observed for prediabetes patients in the validation cohort (P < 0.001) together with an increase in their TC levels (P < 0.001). There were no differences among groups with respect to age, gender, BMI, ALT, or AST, nor were there any differences in TC or LDL levels among the discovery cohorts.

**Table 1:** Baseline patient characteristics in the discovery and validation cohorts.

	Discovery						Validation					
	Control n=35	prediabetes n=31	T2DM n=27	P_1_ value	P_2_ value	P_3_ value	Control n=150	prediabetes n=170	T2DM n=120	P_1_ value	P_2_ value	P_3_ value
Females (%)	25.71	35.48	25.92	**0.985**	**0.793**	**0.490**	43.33%	35.88%	30.83%	**0.1938**	**0.0505**	**0.6206**
Age (years)	49.26 ± 12.21	46.29 ± 13.38	47.71 ± 12.74	**0.829**	**0.874**	**0.576**	45.96 ± 9.79	47.91 ± 10.96	49.98 ± 9.95	**0.1195**	**0.2071**	**0.2044**
FBG (mmol/L)	5.31 ± 0.45	6.37 ± 0.19	8.20 ± 2.27	**0.00001**	**0.00001**	**0.001**	5.05 ± 0.51	6.43 ± 0.24	9.54 ± 2.57	**0.00001**	**0.00001**	**0.00001**
BMI (kg/m2)	24.42 ± 2.42	24.65 ± 2.40	25.60 ± 3.30	**0.226**	**0.877**	**0.478**	24.80 ± 2.84	25.44 ± 3.36	25.67 ± 4.32	**0.0980**	**0.0619**	**0.7773**
Total cholesterol (mmol/L)	4.53 ± 0.28	4.62 ± 0.34	4.85 ± 0.87	**0.103**	**0.943**	**0.383**	4.70 ± 0.46	4.98 ± 0.73	4.77 ± 0.96	**0.0003**	**0.4920**	**0.1267**
Triglyceride (mmol/L)	1.16 ± 0.25	1.14 ± 0.27	1.59 ± 0.77	**0.013**	**0.821**	**0.017**	1.10 ± 0.28	2.04 ± 1.17	2.07 ± 1.31	**0.00001**	**0.00001**	**0.9590**
HDL-C (mmol/L)	1.45 ± 0.24	1.47 ± 0.25	1.20 ± 0.48	**0.045**	**0.859**	**0.043**	1.43 ± 0.27	1.37 ± 0.27	1.12 ± 0.26	**0.0902**	**0.00001**	**0.00001**
LDL-C (mmol/L)	2.54 ± 0.26	2.66 ± 0.31	2.78 ± 0.72	**0.093**	**0.480**	**0.434**	2.86 ± 0.40	3.01 ± 0.65	3.16 ± 0.78	**0.0400**	**0.0001**	**0.1867**
ALT (U/L)	18.89 ± 5.07	20.00 ± 6.11	24.71 ± 18.86	**0.193**	**0.702**	**0.493**	20.68 ± 6.97	22.87 ± 11.16	23.84 ± 16.56	**0.0780**	**0.0589**	**0.7886**
AST (U/L)	19.54 ± 3.09	18.90 ± 3.87	20.86 ± 11.63	**0.637**	**0.655**	**0.466**	20.54 ± 3.63	20.4 ± 5.40	20.34 ± 9.45	**0.7883**	**0.8071**	**0.9409**

Values are given as mean ± SD or number of individuals (%), unless otherwise indicated. P-value; independent t-test and adjusted by FDR, “P_1_” Control VS Prediabetes, “P_2_” Control VS T2DM, “P_3_” Prediabetes VS T2DM. BMI, body mass index; FBG, fasting blood glucose; HDL-C, high-density lipoprotein cholesterol; LDL-C,low-density lipoprotein cholesterol; ALT, alanine transaminase; AST, aspartate transaminase; NGT, normal glucose tolerance; IFG, impaired fasting glucose; T2DM, type 2 diabetes mellitus. Bold values mean P value.

### 3.2 Reproducibility of the lipidomic analysis

**Figure 1:**
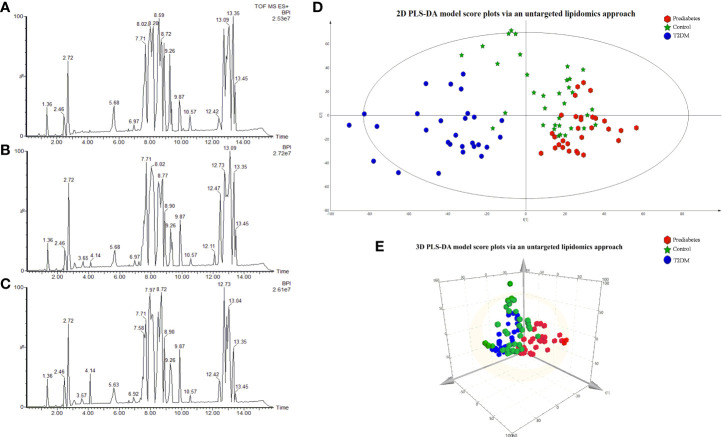
The BPI chromatograms of samples via the untargeted lipidomics approach in the Control group **(A)**; prediabetes group **(B)**; T2DM group **(C)**. 2D PLS-DA model score plots for individual serum samples in the control (green), prediabetes (red), and T2DM (blue) groups analyzed via an untargeted lipidomics approach 3D PCS-DA model score plots **(E)**.

Base peak chromatograms generated in positive ion mode in an untargeted lipidomics analysis are shown in **Figures 1A–C**. To validate the method being used herein for biomarker detection, system stability and result reproducibility were assessed by analyzing pooled QC samples and determining relative standard deviation (RSD%) values corresponding to the peak area for IS compounds (**Table S1**). RSD% values corresponding to the peak area for IS compounds are 6.86%-27.61%. This approach confirmed the high reproducibility and stability of these analyses.

### 3.3 Exploration of distinct lipidomic profiles associated with prediabetes and T2DM

Next, we sought to explore differences in the serum lipidomic profiles of control, prediabetes, and T2DM study subjects by using a PLS-DA model to evaluate the global lipid profiles of these groups as detected through the untargeted lipidomics approach validated above. The resultant 2D and 3D score plots achieved satisfactory classification, revealing that the lipid metabolic state in the serum of prediabetes and T2DM patients was distinct from that in healthy control serum (**Figures 1D, E**). These results suggested that T2DM is associated with the disruption of endogenous metabolic processes such that patients exhibit a distinct metabolic fingerprint. Notably, we also observed substantial separation between prediabetes and T2DM patient samples in these PLS-DA plots, suggesting that prediabetic and diabetic individuals also exhibit distinct lipid metabolic profiled. R^2^ Y represents the goodness of fit of the PLS-DA model on the Y-axis, while Q^2^ estimates predictive capability (60). The R^2^ Yand Q^2^ of the established PLS-D model were 0.925 and 0.609. A permutation test (n=200) was additionally used to validate this model, confirming the goodness of fit and predictive reliability (**Figure S1**).

### 3.4 Identification of putative prediabetes- and T2DM- related biomarkers *via* untargeted and targeted lipidomics analyses

**Figure 2:**
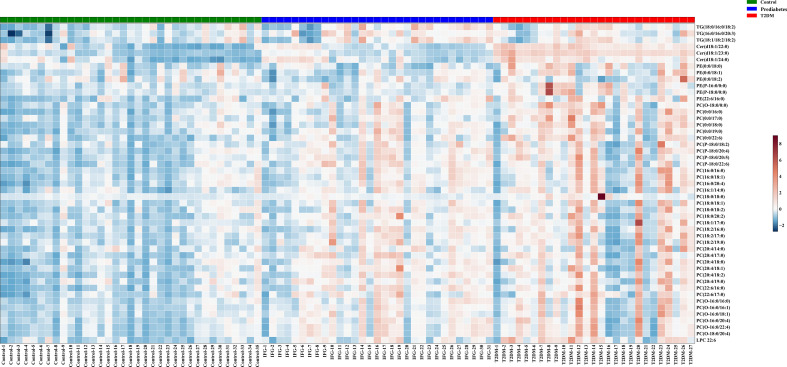
Metabolites that were significantly differentially abundant among groups in the discovery cohort were arranged in a heatmap, with increased and decreased metabolites being shown in red and blue, respectively.

**Figure 3:**
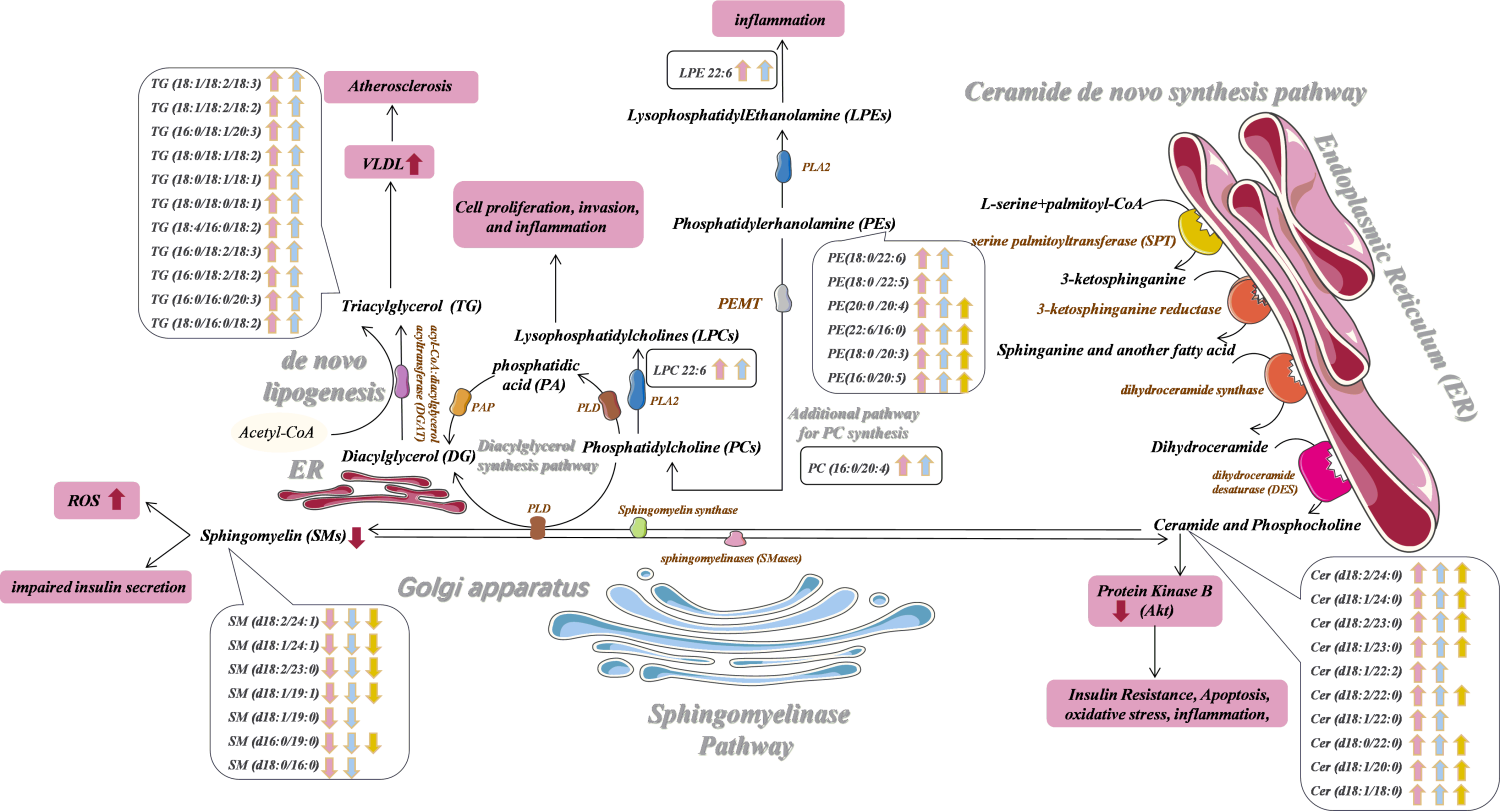
Potential prediabetes and T2DM-related serum biomarker networks. Arrows ("↑↓") indicated metabolites that were significantly up- and down-regulated in prediabetes (pink) and T2DM (blue) patients relative to healthy controls. Metabolites that were significantly altered in the prediabetes group relative to the T2DM group are also shown in yellow. PLD, phospholipase; PAP, phosphatidic acid phosphatase; phospholipase AZ, PLA2; PEMT, phosphatidylethanolamine N-methyltransferase.

**Figure 4:**
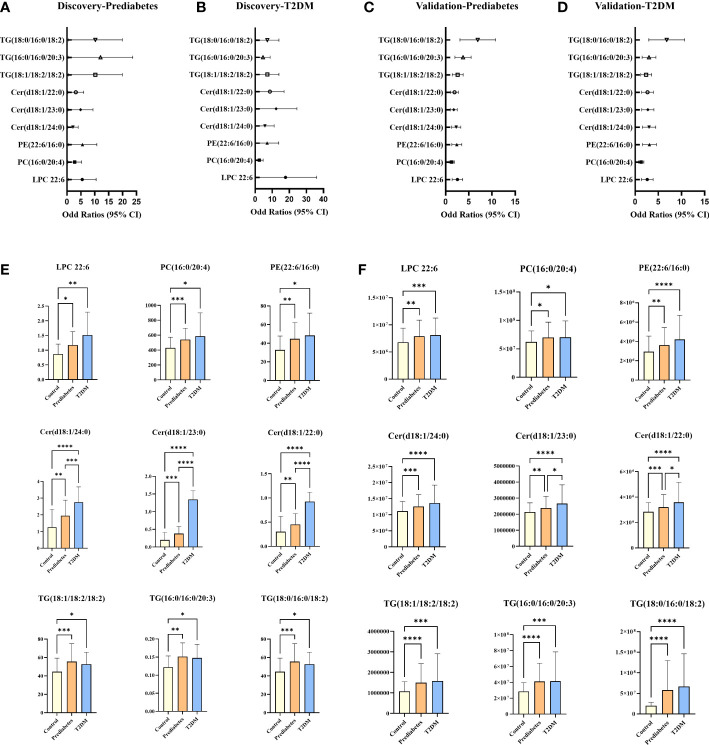
Plot of ORS per one SD increment and 95% Cls of lipids that emerged significant (FDR < 0.05 and P < 0.05) in the discovery and validation cohorts **(A–D)**; potential serum biomarkers in discovery cohort **(E)** and validation cohort **(F)**. *P < 0.05, **P < 0.01, ***P < 0.001, ***P < 0.0001.

For untargeted lipidomics analyses, the Progenesis QI software was used to detect tens of thousands of features in the LC-MS data. Based on ion fragmentation patterns, accurate compound masses, published data, and chemical standards, 166 lipids were identified in these serum samples (**Table S2**). To screen for metabolites that were differentially abundant in the serum of prediabetes and T2DM patients, we next conducted independent sample t-tests with P-value < 0.05 corrected by FDR. 49 candidate lipids show similar significant trends in prediabetes and T2DM relative to controls in untargrted lipidomics analyse (discovery cohort). These differences were additionally emphasized through heatmaps and clustering analyses (**Figure 2**). Based on these results from the discovery cohort, subsequently, a high selectivity, reproducibility and sensitivity targeted lipidomics approach including more than 200 lipids of interest was used to assess the serum lipid profiles of patients in the validation cohort (**Table S3**). In this analysis, 37 lipids including LPCs, LPEs, PCs, PEs, SMs, Cers, and TGs were significantly differentially abundant in samples from the control group and the prediabetes/T2DM groups (**Figure 3**). Levels of all of these lipids were significantly elevated in those with prediabetes/T2DM, suggesting the dysregulation of the ceramide synthesis, SM metabolism, PC biosynthesis pathways (**Figure 3**). By venn diagram (**Figure S2**), 9 potential biomarkers including LPC 22:6, PC(16:0/20:4), PE(22:6/16:0), Cer(d18:1/24:0), Cer(d18:1/23:0), Cer(d18:1/22:0), TG(18:1/18:2/18:2), TG(16:0/16:0/20:3), and TG(18:0/16:0/18:2) (FDR < 0.05 and P < 0.05) were overlapping between 49 candidate lipids metabolites screened from non-targeted lipidomic data (discovery cohort) and 37 differential lipids from targeted lipidomic data (validation cohort), and they show similar significant trends in prediabetes and T2DM relative to controls (**Table 2** and **Figure 4**). A one standard deviation change in the levels of these 9 putative biomarkers was associated with prediabetes and T2DM effect sizes ranging from odds ratios (ORs) of 1.235 - 8.306 and 1.189 - 11.479, respectively (**Figure 4**).

**Table 2:** Potential serum biomarkers.

	Discovery			Validation		
	FDR-adjusted P-value and trend			FDR-adjusted P-value		
Lipid	Control VS Prediabetes	Control VS T2DM	Prediabetes VS T2DM	Control VS Prediabetes	Control VS T2DM	Prediabetes VS T2DM
LPC 22:6	**0.0078 (↑)**	**0.0004 (↑)**	**0.4031 (-)**	**0.0007 (↑)**	**0.00001 (↑)**	**0.7424 (-)**
PC(16:0/20:4)	**0.0081 (↑)**	**0.0181 (↑)**	**0.8881 (-)**	**0.0058 (↑)**	**0.0076 (↑)**	**0.9945 (-)**
PE(22:6/16:0)	**0.0069 (↑)**	**0.0367 (↑)**	**0.7589 (-)**	**0.0008 (↑)**	**0.00001 (↑)**	**0.2894 (-)**
Cer(d18:1/24:0)	**0.0130 (↑)**	**0.0005 (↑)**	**0.0033 (↑)**	**0.0004 (↑)**	**0.00001 (↑)**	**0.2065 (-)**
Cer(d18:1/23:0)	**0.0059 (↑)**	**0.0012 (↑)**	**0.000001 (↑)**	**0.0007 (↑)**	**0.00001 (↑)**	**0.0422 (↑)**
Cer(d18:1/22:0)	**0.0296 (↑)**	**0.0030 (↑)**	**0.000001 (↑)**	**0.0006 (↑)**	**0.00001 (↑)**	**0.0453 (↑)**
TG(18:1/18:2/18:2)	**0.0140 (↑)**	**0.0314 (↑)**	**0.6526 (-)**	**0.00001 (↑)**	**0.0001 (↑)**	**0.8134 (-)**
TG(16:0/16:0/20:3)	**0.0042 (↑)**	**0.0087 (↑)**	**0.6971 (-)**	**0.00001 (↑)**	**0.0003 (↑)**	**0.9209 (-)**
TG(18:0/16:0/18:2)	**0.0122 (↑)**	**0.0054 (↑)**	**0.5711 (-)**	**0.00001 (↑)**	**0.0003 (↑)**	**0.1559 (-)**

P-value corrected by FDR; “↑” means a higher level of metabolites; “↓” means a lower level of metabolites; “–” represents no statistically significant difference Control represents control group; prediabetes represents prediabetes group; T2DM represents T2DM group.

Bold values mean P value.

### 3.5 Integrated biomarker development and validation

**Figure 5:**
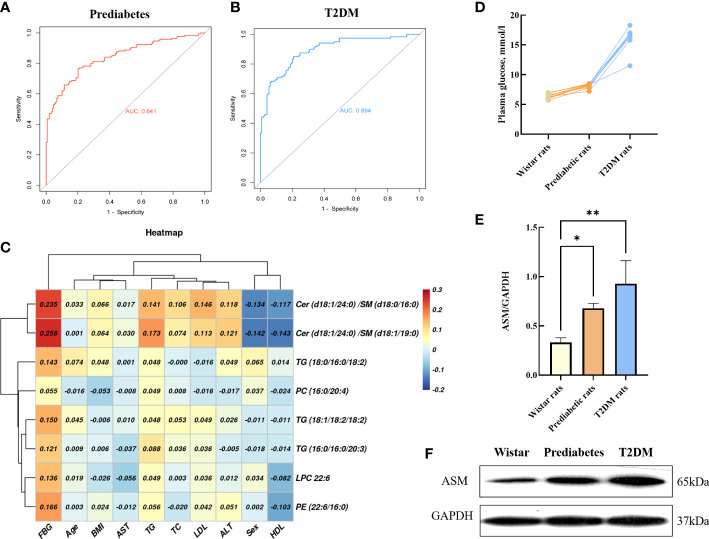
ROC curves of the integrated biomarker in prediabetes group **(A)**, T2DM group **(B)**. Heat map of the Pearson correlation coefficients between potential biomarkers and clinical parameters **(C)**. Basal plasma glucose in GK and control Wistar rats **(D)**. Representative Western blot gel documents and summarized data showed the expression of ASM in adipose tissue **(E, F)**. *P < 0.05, **P < 0.01.

While no significant differences in SMs levels were observed among groups in the discovery cohort, levels of SM (d18:2/24:1), SM (d18:1/24:1), SM (d18:2/23:0), SM (d18:1/19:1), SM (d18:1/19:0), SM (d16:0/19:0) and SM (d18:0/16:0) trended downwards in prediabetes and T2DM samples from the validation cohort (**Figure 4**). Ceramides and SMs are closely linked through the sphingomyelinase pathway, and several ceramide levels trended upward in the prediabetes and T2DM groups in both cohorts. Sphingomyelinase-regulated Cer/SM balance plays a variety of roles in cancer, coronary heart disease and neurodegenerative disorders progression and prevention (16, 61, 62), To investigate whether Cer/SM can predict prediabetes and T2DM, we have carried out binary logistic regression and ROC curve analyses for Cer(d18:1/24:0), Cer(d18:1/23:0), Cer(d18:1/22:0) first. The results show that Cer(d18:1/24:0) have higher predictive power in prediabetes and T2DM compared with Cer(d18:1/23:0) and Cer(d18:1/22:0) (**Figures S3A, B**). Then we performed binary logistic regression and ROC curve analyses for the ratio of Cer(d18:1/24:0) to 7 different SM such as Cer(d18:1/24:0)/SM(d18:2/24:1), Cer(d18:1/24:0)/SM(d18:1/24:1), Cer(d18:1/24:0)/SM(d18:2/23:0), Cer(d18:1/24:0)/SM(d18:1/19:1), Cer(d18:1/24:0)/SM(d18:1/19:0), Cer(d18:1/24:0)/SM(d16:0/19:0) and Cer(d18:1/24:0)/SM(d18:0/16:0). The results show that Cer(d18:1/24:0)/SM(d18:1/19:0) and Cer(d18:1/24:0)/SM(d18:0/16:0) have higher predictive power in prediabetes and T2DM compared with others candidate features (**Figures S3C, D**). As such, we selected Cer(d18:1/24:0)/SM(d18:1/19:0) and Cer(d18:1/24:0)/SM(d18:0/16:0) as candidate features for the development of an integrated diagnostic biosignature for prediabetes and T2DM. The resultant integrated potential biomarker model consisted of LPC 22:6, PC(16:0/20:4), PE(22:6/16:0), Cer(d18:1/24:0)/SM(d18:1/19:0), Cer(d18:1/24:0)/SM(d18:0/16:0), TG(18:1/18:2/18:2), TG(16:0/16:0/20:3), and TG(18:0/16:0/18:2), and was assessed through binary logistic regression and ROC curve analyses. As shown in **Figures 5A, B**, the AUC values for this integrated biomarker in prediabetes and T2DM patients were 0.841 (cutoff: 0.565) and 0.894 (cutoff: 0.633), respectively. As all of these values were > 0.5, this indicated that this model is reliable and able to effectively diagnose prediabetes and T2DM. Pearson correlation analyses were then performed to assess relationships between these biomarkers and clinical parameters, revealing the levels of all of these biomarkers to be positively correlated with patient FBG (**Figure 5C**). We additionally found that Cer(d18:1/24:0)/SM(d18:1/19:0) and Cer(d18:1/24:0)/SM(d18:0/16:0) were significantly negatively correlated with sex (**Figure 5C**). PE(22:6/16:0) and TG (18:0/16:0/18:2) levels were positively correlated with TG. In addition, TG (18:0/16:0/18:2) level was significantly negatively correlated with LDL level, and PE(22:6/16:0) level were significantly negatively correlated with HDL.

### 3.6 Increased ASM protein expression in prediabetes and T2DM rats

As shown in **Figure 5D**, Wistar rats and prediabetic rats had comparable non-fasting blood glucose, and the non-fasting blood glucose values of T2DM rats were about >2 times higher compared to wistar rats. The intensity of individual ASM bands were obtained by western blot analysis of GK rat adipose tissue. Compared with wistar rats, the levels of ASM in prediabetic rats (3-week GK rat) and T2DM rats (12-week GK rat) were significantly increased (p < 0.05) (**Figures 5E, F**), which demonstrated the process of diabetes could affect the changes of ASM content in the patient.

## 4 Discussion

In this study, we employed targeted and untargeted approaches to identify serum lipid profiles in control, prediabetes, and T2DM patients *via* UHPLC-MS and UHPLC-MS/MS. This approach led to the identification of LPC, PC, PE, Cer, SM, and TG lipids that were differentially abundant in those with prediabetes/T2DM relative to control individuals.

Ceramides are the simplest sphingolipid family molecules and are central to sphingolipid metabolism such that they can impact important T2DM-related processes such as insulin resistance, oxidative stress, inflammation, and apoptosis (63, 64). There are three primary ceramide synthesis pathways (65, 66). The first of these involved *de novo* ceramide synthesis within the endoplasmic reticulum (ER) from L-serine and palmitoyl-CoA *via* a multi-stage process (**Figure 3**) (67, 68). Enhanced *de novo* ceramide synthesis can promote protein phosphatase 2A (PPA2) activation, thereby inhibiting insulin sensitivity and β-cell function through the inactivation of protein kinase B (Akt) in the insulin-signaling pathway (69–71). Sphingosine can be used to generate ceramide by many enzymes through a recycling pathway, such as lysosomal ceramidase and ceramide synthetase in the ER (72, 73). Ceramides can also be synthesized through the hydrolysis of SM and glycosphingolipids by sphingomyelinase (SMase) within the Golgi. Through the activity of sphingomyelin synthase (SMS) and phospholipase (PLD), the phosphocholine portion of PC can be transferred to the primary hydroxyl group of ceramide to yield diacylglycerol (DG) and SM, the latter of which is an important bioactive lipid associated with cellular proliferation, migration, and survival (74, 75). We did not detect significant differences in SM levels among groups for serum samples in the discovery cohort. Whereas in the validation cohort, compared with controls, we observed significantly lower levels of SM (d18:2/24:1), SM (d18:1/24:1), SM (d18:2/23:0), SM (d18:1/19:1), SM (d18:1/19:0), SM (d16:0/19:0) and SM (d18:0/16:0) in prediabetes and T2DM patient serum samples. This may suggest that the limited number of samples in the discovery cohort may have yielded false-negative results. We also found that ceramides including Cer(d18:1/24:0), Cer(d18:1/23:0), and Cer(d18:1/22:0) were significantly more abundant in prediabetes and T2DM patients relative to controls in both cohorts. Multiple prior analyses (76, 77), including the European Prospective Investigation into Cancer and Nutrition (EPIC)-Potsdam study (78), have found SM levels to be negatively correlated with T2DM incidence. Similarly, one large cohort analysis of prediabetic and diabetic individuals found that odd-chain SMs were negatively correlated with T2DM risk (27), in line with our findings. We detected significant disruptions in ceramide and SM homeostasis in prediabetes and T2DM patients. This may be the result of the increased expression of enzymes responsible for regulating the conversion between Cer and SM, such as Smases like acid sphingomyelinase (79),. The results of western blot analysis of frozen adipose tissue from 3- and 12-week GK rats also confirmed that ASM is responsible for significant disruptions in ceramide and sphingomyelin homeostasis in prediabetes and T2DM patients. Mice in which SM synthase has been knocked out exhibited reduced SM levels, ceramide accumulation, and impaired mitochondrial activity resulting in impaired ATP production, increased reactive oxygen species (ROS) levels, and decreased glucose-induced insulin secretion, consistent with our hypothesis (80). This ceramide/SM homeostasis has been suggested to be a promising target for therapeutic intervention in multiple pathological contents (81), though whether glucose supplementation can effectively modulate sphingolipid metabolism within β cells by enhancing ceramide to SM conversion remains to be confirmed (82). We ultimately selected Cer(d18:1/24:0)/SM(d18:1/19:0) [(OR: 2.980; 95% CI:1.874-4.737 in prediabetes) and [(OR: 5.507; 95% CI: 3.233-9.379 in T2DM)] and Cer(d18:1/24:0)/SM(d18:0/16:0) [(OR: 2.883; 95% CI:1.801-4.614 in prediabetes) and (OR: 8.308; 95% CI: 4.778-14.445 in T2DM)] as one of components of an integrated biomarker model capable of predicting prediabetes and T2DM risk.

PE synthesis is important in the metabolic processing of lipids in the muscle tissue, and muscle PE levels may be linked to insulin resistance (83). Plasma PE levels have been shown to rise in individuals affected by insulin resistance in population studies (84). In line with such findings, we observed significant increases in PE(22:6/16:0) levels in the serum of prediabetes and T2DM patients in the discovery and verification cohorts. PC is the most common phospholipid in the body, wherein it is produced both by the Kennedy pathway and by additional synthetic pathways in the liver catalyzed by phosphatidylethanolamine N-methyltransferase (PEMT) (85). Samad et al. (86) reported that individuals with diabetes exhibit plasma PC levels distinct from those in healthy individuals. Consistently, we found that PC (16:0/20:4) levels were significantly altered in prediabetes and T2DM patients in both cohorts. Phospholipase A2 (PLA2) can catalyze the formation of LPC from PC (87). LPC is a lipid that serves as an important signaling molecule in the context of cellular proliferation and invasion, and increase levels of LPC 22:6 have previously been reported in obese individuals and those with prediabetes or diabetes (88, 89). The proinflammatory properties of LPC have also been previously documented, as it can both drive inflammatory molecule upregulation (90) and increase vascular endothelial permeability (91). Following PC synthesis through the additional pathway in the liver, PC and ceramide can processed by PLD to yield DG and SM. DG in turn gives rise to TG under the action of acyl-CoA: diacylglycerol acyltransferase (DGAT). Aberrant PC metabolism may increase levels of TG through the activation of SREBP-1 and the induction of *de novo* lipogenesis (92–94). Levels of TG, in turn, are well-studied as a risk factor linked to dysregulated glucose metabolism in the general population. Our Pearson correlation analyses revealed a positive correlation between plasma TG levels and FBG, with plasma TG (18:1/18:2/18:2), TG (16:0/16:0/20:3), and TG (18:0/16:0/18:2) levels being significantly elevated in prediabetes and T2DM patients relative to controls. T2DM patients inevitably exhibit hyperlipidemia, while individuals with prediabetes frequently present with higher circulating TG and free fatty acid (FFA) levels (95), in part owing to impaired lipid processing within adipose tissue (96). Diabetes-related dyslipidemia is also linked with a marked increase in cardiovascular risk (97).

## 5 Conclusions

In this study, we first herein conducted an untargeted lipidomics analysis of newly diagnosed Chinese prediabetic and T2DM patients in a discovery cohort, leading to the identification of changing phospholipid and sphingolipid profiles associated with prediabetes and T2DM that were confirmed for the first time in a separate validation cohort *via* targeted lipidomics analyses. Furthermore, potential biomarkers were confirmed for the first time in separate validation cohort *via* targeted lipidomics analyses. Moreover, the results confirmed ASM is responsible for significant disruptions in ceramide and sphingomyelin homeostasis in prediabetic and T2DM. Finally, this study developing a new integrated biomarker signature that may better aid in the diagnosis of Chinese prediabetes and T2DM, and provides a better biological understanding of the insidious progression to diabetes from a lipid perspective.

## Data availability statement

The raw data supporting the conclusions of this article will be made available by the authors, without undue reservation.

## Ethics statement

The studies involving human participants were reviewed and approved by The Ethics Committee of Scientific Research, Beijing Shijitan Hospital, Capital Medical University. The patients/participants provided their written informed consent to participate in this study. The animal study was reviewed and approved by The China Pharmaceutical University Animal Care and Use Committee.

## Author contributions

NG and DanY conceived the project. NG contributed to the concept development, study design, and edited the manuscript. DanY collected the clinical information, interpreted the data. JY performed the experiments, performed data analysis and wrote the manuscript. DawY, HY and ZW were contributed to the supervision of the experimental process and helped write the manuscript. MW revised the manuscript. All authors contributed to the article and approved the submitted version.
