# Supplementary material for: Integrated lipids biomarker of the prediabetes and type 2 diabetes mellitus Chinese patients
Source: Front Endocrinol (Lausanne). 2023 Jan 20;13:1065665. doi: 10.3389/fendo.2022.1065665 (PMC9897314; doi:10.3389/fendo.2022.1065665)
Supplement: Supplementary file 1 [file DataSheet_1.docx]

Supplementary Material

## Supplementary Figures


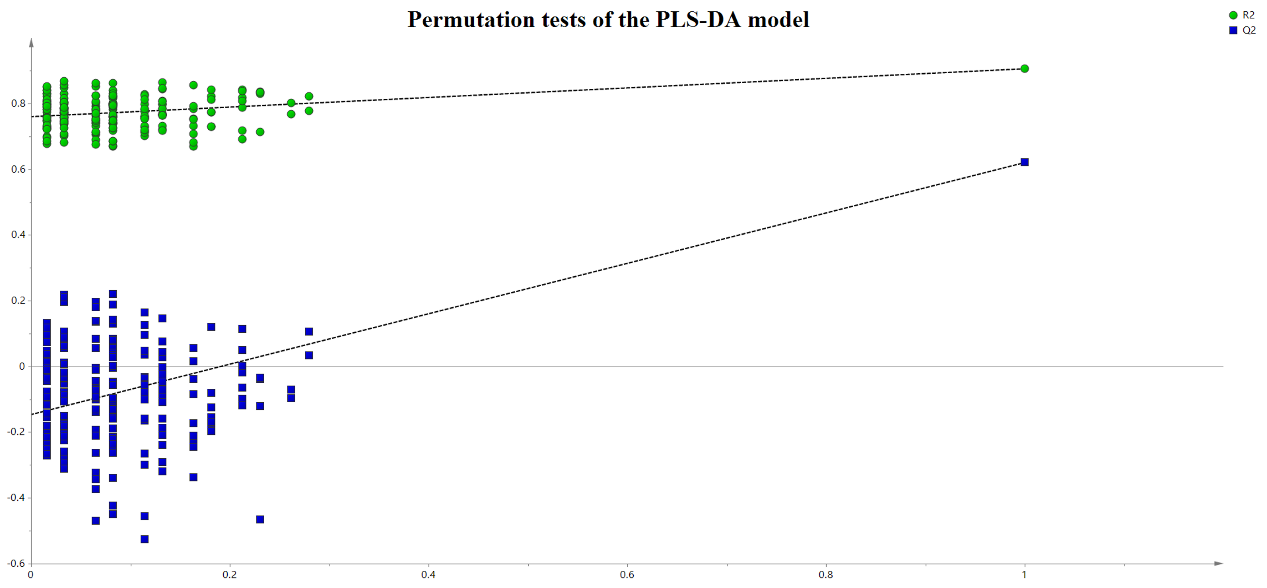


**Figure S1.** Permutation tests (n=200) of the PLS-DA model


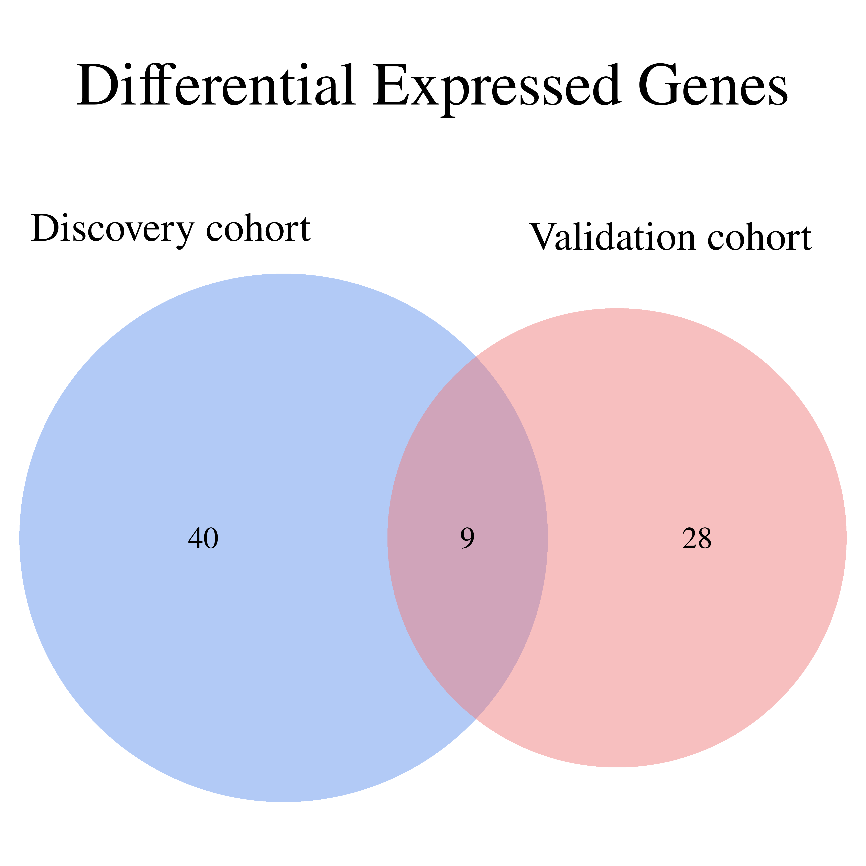


Figure S2. Venn diagram of difference lipids between discovery cohort and validation cohort.


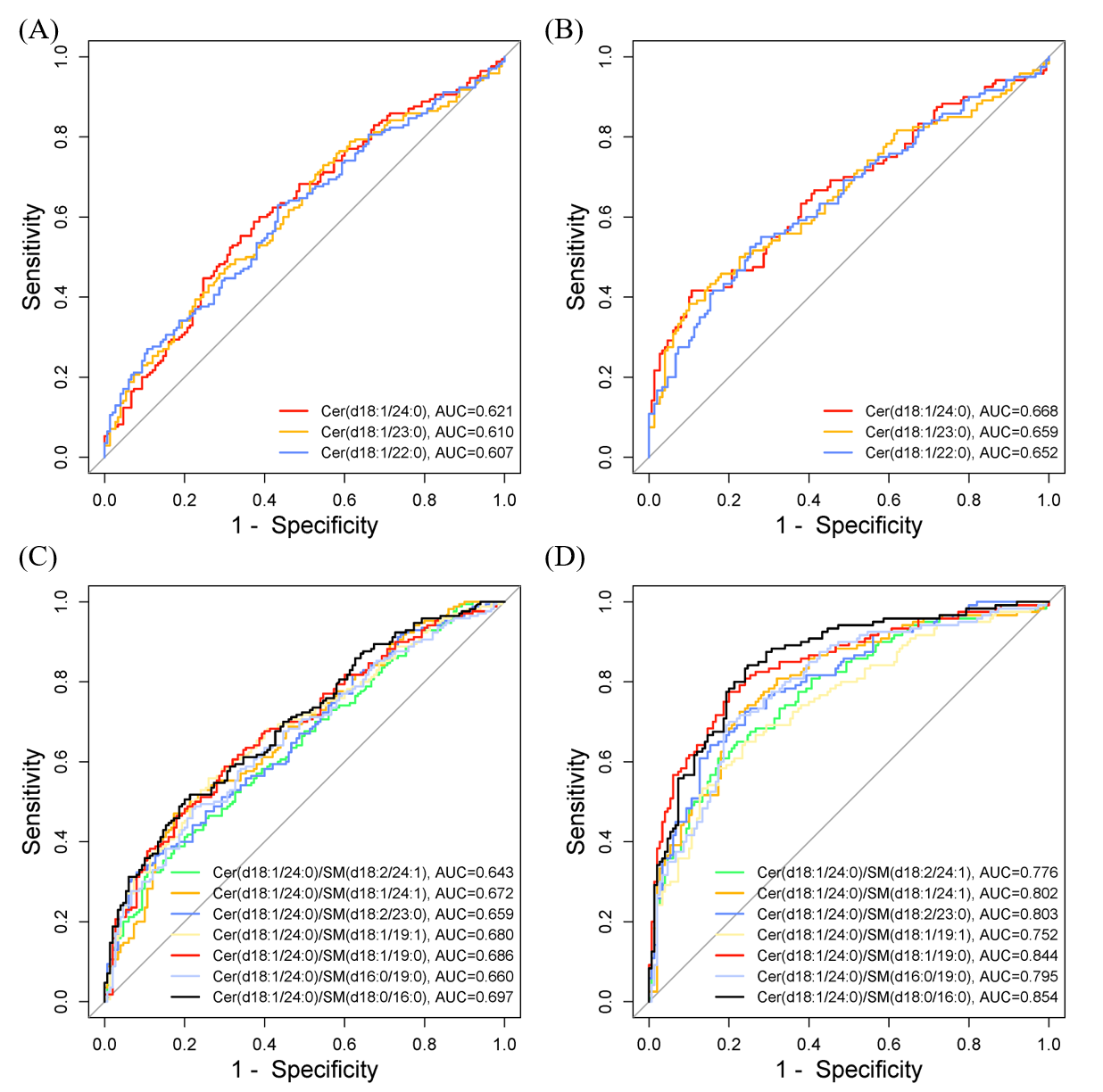


**Figure S3.** ROC curves of the candidate biomarker in prediabetes group (A, C) and T2DM group (B, D).

## Supplementary Tables

**Table S1.** Repeatability results for internal standards in untargeted and targeted lipidomics in serum.

|  |  |  | RSD% | |
| --- | --- | --- | --- | --- |
| RT(min) | Compound | Ion mode | Untargeted lipidomics | Targeted lipidomics |
| 4.01 | LPC 19:0 | ESI (+) | 16.53 | 8.62 |
| 5.81 | PE(12:0/13:0) | ESI (+) | 27.61 | 7.59 |
| 5.94 | SM(d18:1/12:0) | ESI (+) | 6.87 | 8.60 |
| 9.03 | Cer(d18:1/17:0) | ESI (+) | 18.76 | 8.92 |
| 10.36 | PC(19:0/19:0) | ESI (+) | 25.28 | 15.73 |
| 12.73 | TG (15:0/15:0/15:0) | ESI (+) | 19.76 | 6.86 |

**Table S2.** Untargeted lipidomics identification of potential serum biomarkers.

| RT (min) | Ion mode | Assigned identity | Molecular formula | Precursor ion adduct | Exact mass (Da) | Mean measured mass (Da) | Mass Accuracy (ppm) | Product ion of ESI/MS2 |
| --- | --- | --- | --- | --- | --- | --- | --- | --- |
| 1.72 | ESI (+) | PC(0:0/14:0) | C_22_H_46_NO_7_P | [M+H]^+^ | 468.309 | 468.3093 | 0.64 | 184.0754 |
| 1.84 | ESI (+) | LPC 14:0* | C_22_H_46_NO_7_P | [M+H]^+^ | 468.309 | 468.3086 | -0.85 | 184.0746 |
| 1.85 | ESI (+) | PC(0:0/18:3) | C_26_H_48_NO_7_P | [M+H]^+^ | 518.3246 | 518.3273 | 5.21 | 184.0746 |
| 1.92 | ESI (+) | PC(0:0/20:5) | C_28_H_48_NO_7_P | [M+H]^+^ | 542.3246 | 542.3262 | 2.95 | 184.0748 |
| 2.02 | ESI (+) | LPC 18:3* | C_26_H_48_NO_7_P | [M+H]^+^ | 518.3246 | 518.3253 | 1.35 | 184.0745 |
| 2.1 | ESI (+) | LPC 20:5* | C_28_H_48_NO_7_P | [M+H]^+^ | 542.3246 | 542.3258 | 2.21 | 184.0748 |
| 2.1 | ESI (+) | PC(0:0/16:1) | C_24_H_48_NO_7_P | [M+H]^+^ | 494.3246 | 494.3251 | 1.01 | 184.0747 |
| 2.15 | ESI (+) | LPC 16:1* | C_24_H_48_NO_7_P | [M+H]^+^ | 494.3246 | 494.3237 | -1.82 | 184.0739 |
| 2.28 | ESI (+) | PE(0:0/18:2) | C_23_H_44_NO_7_P | [M+H]^+^ | 478.2933 | 478.2938 | 1.05 | 184.0739 |
| 2.33 | ESI (+) | PC(0:0/18:2) | C_26_H_50_NO_7_P | [M+H]^+^ | 520.3403 | 520.3394 | -1.73 | 184.0739 |
| 2.33 | ESI (+) | PE(0:0/22:6) | C_27_H_44_NO_7_P | [M+H]^+^ | 526.2933 | 526.2983 | 9.5 | 184.0738 |
| 2.41 | ESI (+) | PC(0:0/22:6) | C_30_H_50_NO_7_P | [M+H]^+^ | 568.3403 | 568.3411 | 1.41 | 184.074 |
| 2.41 | ESI (+) | PC(0:0/20:4) | C_28_H_50_NO_7_P | [M+H]^+^ | 544.3403 | 544.3394 | -1.65 | 184.0738 |
| 2.46 | ESI (+) | LPC 18:2* | C_26_H_50_NO_7_P | [M+H]^+^ | 520.3403 | 520.3391 | -2.31 | 184.0738 |
| 2.46 | ESI (+) | PE(0:0/16:0) | C_21_H_44_NO_7_P | [M+H]^+^ | 454.2933 | 454.2946 | 2.86 | 184.074 |
| 2.54 | ESI (+) | LPC 22:6* | C_30_H_50_NO_7_P | [M+H]^+^ | 568.3403 | 568.3387 | -2.82 | 184.0738 |
| 2.54 | ESI (+) | LPC 20:4* | C_28_H_50_NO_7_P | [M+H]^+^ | 544.3403 | 544.3389 | -2.57 | 184.0738 |
| 2.54 | ESI (+) | PC(0:0/16:0) | C_24_H_50_NO_7_P | [M+H]^+^ | 496.3403 | 496.3394 | -1.81 | 184.0738 |
| 2.72 | ESI (+) | LPC 16:0* | C_24_H_50_NO_7_P | [M+H]^+^ | 496.3403 | 496.3404 | 0.2 | 184.0739 |
| 2.85 | ESI (+) | PE(0:0/18:1) | C_23_H_46_NO_7_P | [M+H]^+^ | 480.309 | 480.3084 | -1.25 | 184.0739 |
| 2.9 | ESI (+) | PC(0:0/18:1) | C_26_H_52_NO_7_P | [M+H]^+^ | 522.3559 | 522.3548 | -2.11 | 184.0738 |
| 2.9 | ESI (+) | PC(0:0/17:0) | C_25_H_52_NO_7_P | [M+H]^+^ | 510.3559 | 510.3583 | 4.7 | 184.0739 |
| 3.03 | ESI (+) | PE(P-16:0/0:0) | C_21_H_44_NO_6_P | [M+H]^+^ | 438.2984 | 438.2984 | 0 | 184.074 |
| 3.03 | ESI (+) | PC(O-16:0/0:0) | C_24_H_52_NO_6_P | [M+H]^+^ | 482.361 | 482.3597 | -2.7 | 184.074 |
| 3.08 | ESI (+) | LPC 18:1* | C_26_H_52_NO_7_P | [M+H]^+^ | 522.3559 | 522.3551 | -1.53 | 184.074 |
| 3.08 | ESI (+) | PC(P-16:0/0:0) | C_24_H_50_NO_6_P | [M+H]^+^ | 480.3454 | 480.3421 | -6.87 | 184.074 |
| 3.08 | ESI (+) | PC(0:0/22:4) | C_30_H_54_NO_7_P | [M+H]^+^ | 572.3716 | 572.3807 | 15.9 | 184.0739 |
| 3.16 | ESI (+) | PC(0:0/20:2) | C_28_H_54_NO_7_P | [M+H]^+^ | 548.3716 | 548.3744 | 5.11 | 184.0746 |
| 3.21 | ESI (+) | LPC 22:4* | C_30_H_54_NO_7_P | [M+H]^+^ | 572.3716 | 572.3723 | 1.22 | 184.0746 |
| 3.21 | ESI (+) | LPC 17:0* | C_25_H_52_NO_7_P | [M+H]^+^ | 510.3559 | 510.3554 | -0.98 | 184.074 |
| 3.26 | ESI (+) | LPC 20:2 | C_28_H_54_NO_7_P | [M+H]^+^ | 548.3716 | 548.3721 | 0.91 | 184.0751 |
| 3.34 | ESI (+) | PC(P-18:0/0:0) | C_26_H_54_NO_6_P | [M+H]^+^ | 508.3767 | 508.3775 | 1.57 | 184.0751 |
| 3.47 | ESI (+) | PC(0:0/18:0) | C_26_H_54_NO_7_P | [M+H]^+^ | 524.3716 | 524.3702 | -2.67 | 184.074 |
| 3.47 | ESI (+) | PE(0:0/18:0) | C_23_H_48_NO_7_P | [M+H]^+^ | 482.3246 | 482.3243 | -0.62 | 184.074 |
| 3.65 | ESI (+) | LPC 18:0* | C_26_H_54_NO_7_P | [M+H]^+^ | 524.3716 | 524.3708 | -1.53 | 184.074 |
| 3.88 | ESI (+) | PC(O-18:0/0:0) | C_26_H_56_NO_6_P | [M+H]^+^ | 510.3923 | 510.3916 | -1.37 | 184.0749 |
| 4.01 | ESI (+) | PC(0:0/19:0) | C_27_H_56_NO_7_P | [M+H]^+^ | 538.3872 | 538.3865 | -1.3 | 184.0748 |
| 4.32 | ESI (+) | PE(P-18:0/0:0) | C_23_H_48_NO_6_P | [M+H]^+^ | 466.3297 | 466.3284 | -2.79 | 184.0749 |
| 4.44 | ESI (+) | LPC 20:0* | C_28_H_58_NO_7_P | [M+H]^+^ | 552.4029 | 552.4038 | 1.63 | 184.0746 |
| 5.32 | ESI (+) | LPC 22:0* | C_30_H_62_NO_7_P | [M+H]^+^ | 580.4342 | 580.4354 | 2.07 | 184.0746 |
| 6.25 | ESI (+) | LPC 24:0* | C_32_H_66_NO_7_P | [M+H]^+^ | 608.4655 | 608.4653 | -0.33 | 184.0747 |
| 6.79 | ESI (+) | SM(d16:0/16:1)* | C_37_H_75_N_2_O_6_P | [M+H]^+^ | 675.5441 | 675.5433 | -1.18 | 184.074 |
| 6.92 | ESI (+) | PC(22:6/14:0) | C_44_H_76_NO_8_P | [M+H]^+^ | 778.5387 | 778.5391 | 0.51 | 184.074 |
| 6.97 | ESI (+) | SM(d16:1/18:1)* | C_39_H_77_N_2_O_6_P | [M+H]^+^ | 701.5597 | 701.5588 | -1.28 | 184.074 |
| 7.09 | ESI (+) | PC(16:1/14:0) | C_38_H_74_NO_8_P | [M+H]^+^ | 704.523 | 704.5256 | 3.69 | 184.074 |
| 7.22 | ESI (+) | SM(d16:1/17:0) | C_38_H_77_N_2_O_6_P | [M+H]^+^ | 689.5597 | 689.5587 | -1.45 | 184.0739 |
| 7.22 | ESI (+) | PE(16:0/16:0)* | C_37_H_74_NO_8_P | [M+H]^+^ | 692.523 | 692.5249 | 6.5 | 551.5034 |
| 7.22 | ESI (+) | PC(14:0/18:2) | C_40_H_76_NO_8_P | [M+H]^+^ | 730.5387 | 730.5378 | -1.23 | 184.0739 |
| 7.4 | ESI (+) | PC(20:4/16:1) | C_44_H_78_NO_8_P | [M+H]^+^ | 780.5543 | 780.5541 | -0.26 | 184.0742 |
| 7.4 | ESI (+) | PC(18:3/16:0) | C_42_H_78_NO_8_P | [M+H]^+^ | 756.5543 | 756.5544 | 0.13 | 184.0742 |
| 7.58 | ESI (+) | PE(18:2/18:0) | C_41_H_78_NO_8_P | [M+H]^+^ | 744.5543 | 744.5538 | -0.67 | 603.5336 |
| 7.66 | ESI (+) | SM(d18:1/16:0)* | C_39_H_79_N_2_O_6_P | [M+H]^+^ | 703.5754 | 703.5748 | -0.85 | 184.0757 |
| 7.66 | ESI (+) | PC(16:0/14:0) | C_38_H_76_NO_8_P | [M+H]^+^ | 706.5387 | 706.5545 | 22.36 | 184.0755 |
| 7.71 | ESI (+) | PC(22:6/16:0)／PC(20:4/18:2) | C_46_H_80_NO_8_P | [M+H]^+^ | 806.57 | 806.5719 | 2.36 | 184.0756 |
| 7.79 | ESI (+) | SM (d18:1/18:1) | C_41_H_81_N_2_O_6_P | [M+H]^+^ | 729.591 | 729.5904 | -0.82 | 184.0741 |
| 7.79 | ESI (+) | PC(20:4/14:0) | C_42_H_76_NO_8_P | [M+H]^+^ | 754.5387 | 754.537 | -2.25 | 184.0743 |
| 7.89 | ESI (+) | SM(d18:0/16:0)* | C_39_H_81_N_2_O_6_P | [M+H]^+^ | 705.591 | 705.5903 | -0.99 | 184.0783 |
| 7.89 | ESI (+) | PE(22:6/16:0)* | C_43_H_74_NO_8_P | [M+H]^+^ | 764.523 | 764.524 | 1.31 | 623.5018 |
| 7.89 | ESI (+) | PC(16:1/16:0) | C_40_H_78_NO_8_P | [M+H]^+^ | 732.5543 | 732.5542 | -0.14 | 184.0743 |
| 7.97 | ESI (+) | PC(20:4/20:3) | C_47_H_82_NO_8_P | [M+H]^+^ | 820.5856 | 820.5862 | 0.73 | 184.0766 |
| 7.97 | ESI (+) | PC(16:0/20:4) | C_44_H_80_NO_8_P | [M+H]^+^ | 782.57 | 782.5733 | 4.22 | 184.0788 |
| 8.02 | ESI (+) | SM(d18:1/17:0) | C_40_H_81_N_2_O_6_P | [M+H]^+^ | 717.591 | 717.5897 | -1.81 | 184.0775 |
| 8.02 | ESI (+) | PE(17:0/17:0)* | C_39_H_78_NO_8_P | [M+H]^+^ | 720.5543 | 720.5554 | 1.53 | 579.5154 |
| 8.02 | ESI (+) | PC(20:4/18:1) | C_46_H_82_NO_8_P | [M+H]^+^ | 808.5856 | 808.5845 | -1.36 | 184.0781 |
| 8.02 | ESI (+) | PC(18:2/16:0) | C_42_H_80_NO_8_P | [M+H]^+^ | 758.57 | 758.5704 | 0.53 | 184.0738 |
| 8.1 | ESI (+) | PE(20:4/16:0)* | C_41_H_74_NO_8_P | [M+H]^+^ | 740.523 | 740.5223 | -0.95 | 599.5024 |
| 8.15 | ESI (+) | PE(18:2/16:0) | C_39_H_74_NO_8_P | [M+H]^+^ | 716.523 | 716.5223 | -0.98 | 575.5024 |
| 8.15 | ESI (+) | PC(22:6/17:0) | C_48_H_86_NO_8_P | [M+H]^+^ | 836.6169 | 836.6078 | -10.88 | 184.0755 |
| 8.15 | ESI (+) | PC(20:3/16:0) | C_44_H_82_NO_8_P | [M+H]^+^ | 784.5856 | 784.5853 | -0.38 | 184.0754 |
| 8.15 | ESI (+) | PE(16:0/20:5)* | C_41_H_72_NO_8_P | [M+H]^+^ | 738.5074 | 738.5085 | 1.49 | 597.4846 |
| 8.15 | ESI (+) | PC(P-18:0/20:5) | C_46_H_82_NO_7_P | [M+H]^+^ | 792.5907 | 792.5895 | -1.51 | 184.0754 |
| 8.2 | ESI (+) | PE(18:0/18:1)* | C_41_H_80_NO_8_P | [M+H]^+^ | 746.57 | 746.5684 | -2.14 | 605.5609 |
| 8.33 | ESI (+) | PC(20:4/17:0) | C_45_H_82_NO_8_P | [M+H]^+^ | 796.5856 | 796.5847 | -1.13 | 184.074 |
| 8.33 | ESI (+) | PC(18:2/17:0) | C_43_H_82_NO_8_P | [M+H]^+^ | 772.5856 | 772.5845 | -1.42 | 184.074 |
| 8.33 | ESI (+) | PC(P-16:0/20:4) | C_44_H_80_NO_7_P | [M+H]^+^ | 766.575 | 766.5735 | -1.96 | 184.0746 |
| 8.41 | ESI (+) | PE(18:2/18:1)* | C_41_H_76_NO_8_P | [M+H]^+^ | 742.5387 | 742.534 | 7.54 | 601.5181 |
| 8.41 | ESI (+) | PC(O-16:0/20:4) | C_44_H_82_NO_7_P | [M+H]^+^ | 768.5907 | 768.5896 | -1.43 | 184.0748 |
| 8.41 | ESI (+) | PC(O-16:0/18:3) | C_42_H_80_NO_7_P | [M+H]^+^ | 742.575 | 742.5739 | -1.48 | 184.0748 |
| 8.46 | ESI (+) | PC(O-16:0/18:2) | C_42_H_82_NO_7_P | [M+H]^+^ | 744.5907 | 744.589 | -2.28 | 184.0752 |
| 8.46 | ESI (+) | PC(16:0/16:0) | C_40_H_80_NO_8_P | [M+H]^+^ | 734.57 | 734.5695 | -0.68 | 184.0749 |
| 8.46 | ESI (+) | PC(P-18:0/22:6) | C_48_H_84_NO_8_P | [M+H]^+^ | 834.6013 | 834.6003 | -1.2 | 184.0749 |
| 8.51 | ESI (+) | SM(d18:1/18:0)* | C_41_H_83_N_2_O_6_P | [M+H]^+^ | 731.6067 | 731.6055 | -1.64 | 184.0758 |
| 8.51 | ESI (+) | PC(16:0/18:1) | C_42_H_82_NO_8_P | [M+H]^+^ | 760.5856 | 760.5858 | 0.26 | 184.0756 |
| 8.51 | ESI (+) | PC(P-18:0/20:4) | C_46_H_84_NO_7_P | [M+H]^+^ | 794.6063 | 794.6047 | -2.01 | 184.0761 |
| 8.64 | ESI (+) | SM(d18:2/20:0) | C_43_H_85_N_2_O_6_P | [M+H]^+^ | 757.6223 | 757.6212 | -1.45 | 184.0767 |
| 8.64 | ESI (+) | PE(18:0/22:6)* | C_45_H_78_NO_8_P | [M+H]^+^ | 792.5543 | 792.5613 | 8.83 | 651.5325 |
| 8.72 | ESI (+) | PE(16:0/18:1) | C_39_H_76_NO_8_P | [M+H]^+^ | 718.5387 | 718.5417 | 4.18 | 577.5179 |
| 8.72 | ESI (+) | PC(20:4/18:0) | C_46_H_84_NO_8_P | [M+H]^+^ | 810.6013 | 810.6013 | 0 | 184.0777 |
| 8.72 | ESI (+) | PC(18:0/18:2) | C_44_H_84_NO_8_P | [M+H]^+^ | 786.6013 | 786.6016 | 0.38 | 184.0768 |
| 8.72 | ESI (+) | Cer(d18:1/16:0)* | C_34_H_67_NO_3_ | [M+H]^+^ | 538.5199 | 538.5173 | 3.31 | 264.2688 |
| 8.78 | ESI (+) | PC(18:0/22:5) | C_46_H_88_NO_8_P | [M+H]^+^ | 814.6326 | 814.6226 | -12.28 | 184.0739 |
| 8.82 | ESI (+) | PC(O-16:0/16:1) | C_40_H_80_NO_7_P | [M+H]^+^ | 718.575 | 718.5725 | -3.48 | 184.0735 |
| 8.9 | ESI (+) | PE(20:4/18:0)* | C_43_H_78_NO_8_P | [M+H]^+^ | 768.5543 | 768.5544 | 0.13 | 627.533 |
| 8.9 | ESI (+) | PC(O-16:0/22:4) | C_46_H_86_NO_7_P | [M+H]^+^ | 796.622 | 796.618 | -5.02 | 184.0738 |
| 8.9 | ESI (+) | PC(O-16:0/18:1) | C_42_H_84_NO_7_P | [M+H]^+^ | 746.6063 | 746.6043 | -2.68 | 184.0739 |
| 8.9 | ESI (+) | PC(O-16:0/16:0) | C_40_H_82_NO_7_P | [M+H]^+^ | 720.5907 | 720.5891 | -2.22 | 184.0738 |
| 8.9 | ESI (+) | PC(22:6/18:0) | C_47_H_86_NO_8_P | [M+H]^+^ | 824.6169 | 824.6188 | 2.3 | 184.074 |
| 8.9 | ESI (+) | PC(18:0/20:3) | C_46_H_86_NO_8_P | [M+H]^+^ | 812.6169 | 812.616 | -1.11 | 184.0739 |
| 8.9 | ESI (+) | Cer(d18:0/14:0) | C_32_H_65_NO_3_ | [M+H]^+^ | 512.5042 | 512.5042 | 0 | 266.2788 |
| 8.95 | ESI (+) | PC(18:1/17:0) | C_43_H_84_NO_8_P | [M+H]^+^ | 774.6013 | 774.6 | -1.68 | 184.0745 |
| 8.97 | ESI (+) | SM(d18:1/19:0)* | C_42_H_85_N_2_O_6_P | [M+H]^+^ | 745.6223 | 745.6205 | -2.41 | 184.0739 |
| 9.08 | ESI (+) | PC(18:2/19:0) | C_45_H_86_NO_8_P | [M+H]^+^ | 800.6169 | 800.6148 | -2.62 | 184.0737 |
| 9.08 | ESI (+) | PC(18:0/16:0) | C_42_H_84_NO_8_P | [M+H]^+^ | 762.6013 | 762.5999 | -1.84 | 184.0737 |
| 9.13 | ESI (+) | PC(O-18:0/20:4) | C_46_H_86_NO_7_P | [M+H]^+^ | 796.622 | 796.621 | -1.26 | 184.0737 |
| 9.13 | ESI (+) | PC(P-18:0/18:2) | C_44_H_84_NO_7_P | [M+H]^+^ | 770.6063 | 770.6027 | -4.67 | 184.0737 |
| 9.13 | ESI (+) | PC(P-18:0/18:1) | C_44_H_86_NO_7_P | [M+H]^+^ | 772.622 | 772.6194 | -3.37 | 184.0737 |
| 9.26 | ESI (+) | PC(20:4/19:0) | C_46_H_90_NO_8_P | [M+H]^+^ | 816.6482 | 816.64 | -10.04 | 184.074 |
| 9.26 | ESI (+) | PC(18:0/18:1) | C_44_H_86_NO_8_P | [M+H]^+^ | 788.6169 | 788.6167 | -0.25 | 184.0739 |
| 9.26 | ESI (+) | PE(P-16:0/22:4) | C_43_H_78_NO_7_P | [M+H]^+^ | 752.5594 | 752.5577 | -2.26 | 184.0739 |
| 9.34 | ESI (+) | SM(d18:1/20:0) | C_43_H_87_N_2_O_6_P | [M+H]^+^ | 759.638 | 759.6374 | -0.79 | 184.0739 |
| 9.34 | ESI (+) | PE(P-18:0/18:2) | C_41_H_78_NO_7_P | [M+H]^+^ | 728.5594 | 728.558 | -1.92 | 184.0739 |
| 9.39 | ESI (+) | SM(d18:2/24:1) | C_47_H_91_N_2_O_6_P | [M+H]^+^ | 811.6692 | 811.6689 | -0.37 | 184.0739 |
| 9.39 | ESI (+) | SM(d16:1/24:1)* | C_45_H_89_N_2_O_6_P | [M+H]^+^ | 785.6536 | 785.6531 | -0.64 | 184.0739 |
| 9.39 | ESI (+) | Cer(d18:2/20:0)* | C_38_H_73_NO_3_ | [M+H]^+^ | 592.5668 | 592.5613 | -9.28 | 262.2537 |
| 9.44 | ESI (+) | Cer(d18:1/18:0)* | C_36_H_71_NO_3_ | [M+H]^+^ | 566.5512 | 566.5538 | 3.07 | 264.2698 |
| 9.44 | ESI (+) | Cer(d18:0/18:0)* | C_36_H_73_NO_3_ | [M+H]^+^ | 568.5668 | 568.5764 | 16.88 | 266.2853 |
| 9.57 | ESI (+) | PC(O-18:0/16:0) | C_42_H_86_NO_7_P | [M+H]^+^ | 748.622 | 748.6217 | -0.4 | 184.0739 |
| 9.67 | ESI (+) | SM(d19:1/20:0)* | C_44_H_89_N_2_O_6_P | [M+H]^+^ | 773.6536 | 773.6518 | -2.5 | 184.0737 |
| 9.69 | ESI (+) | Cer(d18:0/16:0)* | C_34_H_69_NO_3_ | [M+H]^+^ | 540.5355 | 540.5356 | 0.19 | 266.2791 |
| 9.75 | ESI (+) | SM(d17:1/24:1) | C_46_H_91_N_2_O_6_P | [M+H]^+^ | 799.6693 | 799.6689 | -0.5 | 184.0739 |
| 9.75 | ESI (+) | PC(18:0/18:0) | C_44_H_88_NO_8_P | [M+H]^+^ | 790.6326 | 790.6326 | 0 | 184.0737 |
| 9.8 | ESI (+) | SM(d18:2/23:0) | C_46_H_91_N_2_O_6_P | [M+H]^+^ | 799.6693 | 799.6689 | -0.5 | 184.0737 |
| 9.87 | ESI (+) | SM(d18:1/24:1)* | C_47_H_93_N_2_O_6_P | [M+H]^+^ | 813.6849 | 813.6849 | 0 | 184.0746 |
| 9.87 | ESI (+) | SM(d18:1/22:0) | C_45_H_91_N_2_O_6_P | [M+H]^+^ | 787.6693 | 787.669 | -0.38 | 184.0746 |
| 10 | ESI (+) | Cer(d18:1/20:0)* | C_38_H_75_NO_3_ | [M+H]^+^ | 596.5981 | 596.5967 | -2.35 | 264.2701 |
| 10.13 | ESI (+) | SM(d17:1/26:1) | C_48_H_95_N_2_O_6_P | [M+H]^+^ | 827.7006 | 827.7009 | 0.36 | 184.0739 |
| 10.13 | ESI (+) | Cer(d18:0/20:0)* | C_38_H_77_NO_3_ | [M+H]^+^ | 596.5981 | 596.6045 | 10.73 | 266.2795 |
| 10.13 | ESI (+) | Cer(d16:1/24:1)／Cer(d18:1/22:1) | C_40_H_77_NO_3_ | [M+H]^+^ | 620.5981 | 620.5984 | 0.48 | 236.2402 |
| 10.13 | ESI (+) | Cer(d18:2/24:1)* | C_42_H_79_NO_3_ | [M+H]^+^ | 646.6138 | 646.6136 | -0.31 | 262.2543 |
| 10.26 | ESI (+) | SM(d17:1/24:0) | C_46_H_93_N_2_O_6_P | [M+H]^+^ | 801.6849 | 801.6853 | 0.5 | 184.0738 |
| 10.49 | ESI (+) | SM(d18:1/26:1) | C_49_H_97_N_2_O_6_P | [M+H]^+^ | 841.7162 | 841.7155 | -0.83 | 184.0738 |
| 10.49 | ESI (+) | Cer(d18:2/23:0) | C_41_H_79_NO_3_ | [M+H]^+^ | 634.6138 | 634.6144 | 0.95 | 262.2498 |
| 10.57 | ESI (+) | SM(d18:1/24:0) | C_47_H_95_N_2_O_6_P | [M+H]^+^ | 815.7006 | 815.7004 | -0.25 | 184.0737 |
| 10.67 | ESI (+) | Cer(d18:1/22:0)* | C_40_H_79_NO_3_ | [M+H]^+^ | 622.6138 | 622.6131 | -1.12 | 264.2691 |
| 10.67 | ESI (+) | Cer(d16:1/24:0)* | C_40_H_79_NO_3_ | [M+H]^+^ | 622.6138 | 622.6135 | -0.48 | 236.2352 |
| 10.8 | ESI (+) | SM(d19:0/24:1) | C_48_H_97_N_2_O_6_P | [M+H]^+^ | 829.7163 | 829.717 | 0.84 | 184.074 |
| 10.8 | ESI (+) | Cer(d18:1/24:1) | C_42_H_81_NO_3_ | [M+H]^+^ | 648.6294 | 648.6284 | -1.54 | 264.2694 |
| 10.8 | ESI (+) | Cer(d18:2/24:0)* | C_42_H_81_NO_3_ | [M+H]^+^ | 648.6294 | 648.6285 | -1.39 | 262.2539 |
| 10.93 | ESI (+) | Cer(d18:0/22:0)* | C_40_H_81_NO_3_ | [M+H]^+^ | 624.6259 | 624.636 | 16.17 | 266.2804 |
| 10.98 | ESI (+) | Cer(d18:1/23:0) | C_41_H_81_NO_3_ | [M+H]^+^ | 636.6294 | 636.6202 | 9.64 | 264.2696 |
| 11.24 | ESI (+) | Cer(18:0/24:0)* | C_42_H_85_NO_3_ | [M+H]^+^ | 652.6602 | 652.6631 | 4.43 | 266.2863 |
| 11.24 | ESI (+) | Cer(d18:1/24:0)* | C_42_H_83_NO_3_ | [M+H]^+^ | 650.6451 | 650.6437 | -2.15 | 264.269 |
| 11.24 | ESI (+) | Cer(d18:0/24:1) | C_42_H_83_NO_3_ | [M+H]^+^ | 650.6451 | 650.6438 | -2 | 266.2809 |
| 11.37 | ESI (+) | Cer(d18:1/26:1)/Cer(d18:2/26:0) | C_44_H_85_NO_3_ | [M+H]^+^ | 676.6607 | 676.6582 | -3.69 | 264.2709 |
| 11.73 | ESI (+) | Cer(d20:1/24:0)* | C_44_H_87_NO_3_ | [M+H]^+^ | 678.6764 | 678.6762 | -0.29 | 310.312 |
| 11.8 | ESI (+) | Cer(d18:1/26:0)* | C_44_H_87_NO_3_ | [M+H]^+^ | 678.6764 | 678.6754 | -1.47 | 264.2694 |
| 12.11 | ESI (+) | TG(18:4/16:0/18:2) | C_55_H_94_O_6_ | [M+NH_4_]^+^ | 868.7394 | 868.7397 | 0.35 | 575.5012 |
| 12.34 | ESI (+) | TG(16:0/16:1/18:3) | C_53_H_94_O_6_ | [M+NH_4_]^+^ | 844.7394 | 844.7394 | 0 | 571.4711 |
| 12.42 | ESI (+) | TG(16:0/18:2/18:3) | C_55_H_96_O_6_ | [M+NH_4_]^+^ | 870.7551 | 870.7562 | 1.26 | 597.4869 |
| 12.42 | ESI (+) | TG(16:0/14:0/18:3)* | C_51_H_92_O_6_ | [M+NH_4_]^+^ | 818.7238 | 818.7233 | -0.61 | 545.4564 |
| 12.47 | ESI (+) | TG(18:1/18:2/18:3) | C_57_H_98_O_6_ | [M+NH_4_]^+^ | 896.7707 | 896.7721 | 1.56 | 597.4869 |
| 12.65 | ESI (+) | TG(16:1/14:0/18:1) | C_51_H_94_O_6_ | [M+NH_4_]^+^ | 820.7394 | 820.7394 | 0 | 521.4556 |
| 12.73 | ESI (+) | TG(16:0/18:2/18:2) | C_55_H_98_O_6_ | [M+NH_4_]^+^ | 872.7707 | 872.7737 | 3.44 | 575.5 |
| 12.73 | ESI (+) | TG(16:0/16:2/18:1) | C_53_H_96_O_6_ | [M+NH_4_]^+^ | 846.7551 | 846.7557 | 0.71 | 549.4865 |
| 12.78 | ESI (+) | TG(18:1/18:2/18:2) | C_57_H_100_O_6_ | [M+NH_4_]^+^ | 898.7864 | 898.7885 | 2.34 | 601.5179 |
| 12.96 | ESI (+) | TG(16:0/14:0/18:1) | C_51_H_96_O_6_ | [M+NH_4_]^+^ | 822.7551 | 822.7554 | 0.36 | 523.4716 |
| 13.07 | ESI (+) | TG(16:0/16:1/18:1) | C_53_H_98_O_6_ | [M+NH_4_]^+^ | 848.7707 | 848.7719 | 1.41 | 549.4873 |
| 13.09 | ESI (+) | TG(16:0/16:0/20:3) | C_55_H_100_O_6_ | [M+NH_4_]^+^ | 874.7864 | 874.7896 | 3.66 | 603.5322 |
| 13.14 | ESI (+) | TG(16:0/18:1/20:3)* | C_57_H_102_O_6_ | [M+NH_4_]^+^ | 900.802 | 900.8037 | 1.89 | 627.5336 |
| 13.27 | ESI (+) | TG(16:0/16:0/18:1) | C_53_H_100_O_6_ | [M+NH_4_]^+^ | 850.7864 | 850.7872 | 0.94 | 577.5201 |
| 13.27 | ESI (+) | TG(18:0/14:0/16:0)* | C_51_H_98_O_6_ | [M+NH_4_]^+^ | 824.7707 | 824.7712 | 0.61 | 523.4731 |
| 13.35 | ESI (+) | TG(18:0/18:1/18:2) | C_57_H_104_O_6_ | [M+NH_4_]^+^ | 902.8177 | 902.8196 | 2.1 | 601.518 |
| 13.35 | ESI (+) | TG(18:0/18:1/18:1) | C_57_H_106_O_6_ | [M+NH_4_]^+^ | 904.8333 | 904.8259 | -8.18 | 603.5345 |
| 13.35 | ESI (+) | TG(18:0/16:0/18:2)* | C_55_H_102_O_6_ | [M+NH_4_]^+^ | 876.802 | 876.8039 | 2.17 | 575.5038 |
| 13.45 | ESI (+) | TG(18:0/18:0/18:1) | C_57_H_108_O_6_ | [M+NH_4_]^+^ | 906.849 | 906.843 | -6.62 | 605.549 |
| 13.45 | ESI (+) | TG(18:0/16:0/16:0) | C_53_H_102_O_6_ | [M+NH_4_]^+^ | 852.802 | 852.8026 | 0.7 | 579.5333 |


* identified with represented standards; # internal standards

**Table S3.** Details for all lipids in identified in targeted lipidomics analyses.

| RT(min) | Compounds | Precursor Ion | Product Ion | Cone(V) | Collision (V) |
| --- | --- | --- | --- | --- | --- |
| 1.67 | LPC18:4 | 516.4 | 184.1 | 46 | 30 |
| 1.72 | LPC 16:2 | 492.3 | 184.1 | 46 | 30 |
| 1.92 | LPC 18:3* | 518.3 | 184.1 | 46 | 30 |
| 1.99 | LPC 20:5* | 542.3 | 184.1 | 46 | 30 |
| 2.06 | LPC 16:1* | 494.3 | 184.1 | 46 | 30 |
| 2.17 | LPC O-16:0 | 482.4 | 184.1 | 46 | 30 |
| 2.18 | LPC 15:0 | 482.3 | 184.1 | 46 | 30 |
| 2.39 | LPC 18:2* | 520.3 | 184.1 | 46 | 30 |
| 2.42 | LPC 22:6* | 568.3 | 184.1 | 46 | 30 |
| 2.45 | LPC 20:4* | 544.3 | 184.1 | 46 | 30 |
| 2.5 | LPC 17:1 | 508.3 | 184.1 | 46 | 30 |
| 2.63 | LPC 16:0* | 496.3 | 184.1 | 46 | 30 |
| 2.75 | LPC 20:3 | 546.4 | 184.1 | 46 | 30 |
| 2.91 | LPC 22:5 | 570.4 | 184.1 | 46 | 30 |
| 2.97 | LPC 18:1* | 522.4 | 184.1 | 46 | 30 |
| 3.13 | LPC 17:0* | 510.4 | 184.1 | 46 | 30 |
| 3.13 | LPC O-18:0 | 510.4 | 184.1 | 46 | 30 |
| 3.14 | LPC 22:4* | 572.4 | 184.1 | 46 | 30 |
| 3.22 | LPC 20:2 | 548.4 | 184.1 | 46 | 30 |
| 3.37 | LPC 19:1 | 536.4 | 184.1 | 46 | 30 |
| 3.57 | LPC 17:2 | 506.3 | 184.1 | 46 | 30 |
| 3.57 | LPC 18:0* | 524.4 | 184.1 | 46 | 30 |
| 3.77 | LPC 20:1 | 550.4 | 184.1 | 46 | 30 |
| 4.07 | LPC 19:0# | 538.3 | 184.1 | 46 | 30 |
| 4.46 | LPC 20:0* | 552.4 | 184.1 | 46 | 30 |
| 4.6 | LPC 22:1 | 578.4 | 184.1 | 46 | 30 |
| 4.95 | LPC 21:0 | 566.4 | 184.1 | 46 | 30 |
| 5.43 | LPC 22:0* | 580.4 | 184.1 | 46 | 30 |
| 5.49 | LPC 24:1 | 606.5 | 184.1 | 46 | 30 |
| 5.9 | PE(12:0/13:0)# | 594.4 | 453.4 | 18 | 20 |
| 6.07 | SM(d18:1/12:0)# | 647.5 | 184.1 | 50 | 30 |
| 6.16 | PC(10:0/16:0) | 650.5 | 184.1 | 74 | 28 |
| 6.21 | SM(d16:1/16:1)* | 673.5 | 184.1 | 50 | 30 |
| 6.32 | LPC 24:0* | 608.5 | 184.1 | 46 | 30 |
| 6.49 | SM(d16:0/15:1) | 661.5 | 184.1 | 50 | 30 |
| 6.66 | SM(d18:1/15:1)* | 687.5 | 184.1 | 50 | 30 |
| 6.74 | PC(18:3/16:2)* | 752.5 | 184.1 | 74 | 28 |
| 6.75 | PC(20:5/22:6) | 852.6 | 184.1 | 74 | 28 |
| 6.97 | SM(d16:1/16:0) | 675.5 | 184.1 | 50 | 30 |
| 7 | PC(12:0/16:0)* | 678.5 | 184.1 | 74 | 28 |
| 7.06 | PC(22:6/14:0) | 778.5 | 184.1 | 74 | 28 |
| 7.11 | SM(d16:1/18:1)* | 701.6 | 184.1 | 50 | 30 |
| 7.29 | PC(20:4/22:6)* | 854.6 | 184.1 | 74 | 28 |
| 7.3 | PC(18:2/20:5)* | 804.6 | 184.1 | 74 | 28 |
| 7.31 | PC(20:4/14:0) | 754.5 | 184.1 | 74 | 28 |
| 7.34 | PC(14:0/18:2) | 730.5 | 184.1 | 74 | 28 |
| 7.36 | PC(20:4/20:4) | 830.6 | 184.1 | 74 | 28 |
| 7.38 | LPC 26:0 | 636.5 | 184.1 | 46 | 30 |
| 7.38 | SM(d16:0/20:3)* | 727.6 | 184.1 | 50 | 30 |
| 7.39 | PC(10:0/19:1) | 690.5 | 184.1 | 74 | 28 |
| 7.39 | SM(d16:0/17:0)* | 691.6 | 184.1 | 50 | 30 |
| 7.39 | SM(d16:1/17:0) | 689.6 | 184.1 | 50 | 30 |
| 7.46 | PC(15:0/22:6) | 792.6 | 184.1 | 74 | 28 |
| 7.53 | PC(20:3/22:6)* | 856.6 | 184.1 | 74 | 28 |
| 7.54 | PC(20:4/16:1) | 780.6 | 184.1 | 74 | 28 |
| 7.57 | SM(d18:1/17:1)* | 715.6 | 184.1 | 50 | 30 |
| 7.58 | PC(18:3/16:0) | 756.6 | 184.1 | 74 | 28 |
| 7.58 | SM(d16:0/22:3)* | 755.6 | 184.1 | 50 | 30 |
| 7.63 | SM(d20:0/22:6) | 805.6 | 184.1 | 50 | 30 |
| 7.71 | PC(15:0/20:4) | 768.6 | 184.1 | 74 | 28 |
| 7.75 | PC(15:0/18:2)* | 744.6 | 184.1 | 74 | 28 |
| 7.77 | PC(13:0/20:4) | 740.5 | 184.1 | 74 | 28 |
| 7.78 | PC(16:1/18:2)* | 756.6 | 184.1 | 74 | 28 |
| 7.8 | SM(d18:1/16:0)* | 703.6 | 184.1 | 50 | 30 |
| 7.81 | PC(16:1/14:0) | 704.5 | 184.1 | 74 | 28 |
| 7.82 | PE(14:0/22:6) | 736.5 | 595.5 | 18 | 20 |
| 7.83 | PC(22:6/16:0) | 806.6 | 184.1 | 74 | 28 |
| 7.85 | LPC 25:0 | 622.5 | 184.1 | 46 | 30 |
| 7.85 | PC(20:4/18:2) | 806.6 | 184.1 | 74 | 28 |
| 7.85 | SM(d20:0/22:5) | 807.6 | 184.1 | 50 | 30 |
| 7.86 | Cer(d18:1/14:0) | 510.5 | 264.3 | 26 | 32 |
| 7.88 | PC(16:2/20:5) | 776.5 | 184.1 | 74 | 28 |
| 7.88 | PE(16:0/16:0)* | 692.5 | 551.5 | 18 | 20 |
| 7.9 | PC(17:1/18:2)* | 770.6 | 184.1 | 74 | 28 |
| 7.99 | PC(16:1/16:0) | 732.6 | 184.1 | 74 | 28 |
| 7.99 | PC(16:1/16:2) | 728.5 | 184.1 | 74 | 28 |
| 7.99 | PC(20:4/20:3) | 832.6 | 184.1 | 74 | 28 |
| 7.99 | SM(d18:1/18:1) | 729.6 | 184.1 | 50 | 30 |
| 8.03 | PC(18:4/22:6) | 826.5 | 184.1 | 74 | 28 |
| 8.03 | PE(22:6/16:0)* | 764.5 | 623.5 | 18 | 20 |
| 8.06 | PC(20:2/22:6) | 858.6 | 184.1 | 74 | 28 |
| 8.1 | PC(16:0/20:4) | 782.6 | 184.1 | 74 | 28 |
| 8.11 | PC(16:0/14:0) | 706.5 | 184.1 | 74 | 28 |
| 8.11 | SM(d18:0/16:0)* | 705.6 | 184.1 | 50 | 30 |
| 8.14 | PC(18:2/16:0) | 758.6 | 184.1 | 74 | 28 |
| 8.2 | PC(15:0/16:0)* | 720.6 | 184.1 | 74 | 28 |
| 8.24 | PC(22:6/17:0) | 820.6 | 184.1 | 74 | 28 |
| 8.24 | SM(d18:1/17:0) | 717.6 | 184.1 | 50 | 30 |
| 8.25 | PC(18:0/20:5)* | 808.6 | 184.1 | 74 | 28 |
| 8.25 | PC(20:4/18:1) | 808.6 | 184.1 | 74 | 28 |
| 8.28 | PE(20:4/16:0)* | 740.5 | 599.5 | 18 | 20 |
| 8.32 | PC(10:0/19:0) | 692.5 | 184.1 | 74 | 28 |
| 8.33 | PE(16:0/20:5)* | 738.5 | 597.5 | 18 | 20 |
| 8.33 | PE(18:2/16:0) | 716.5 | 575.5 | 18 | 20 |
| 8.35 | PC(20:3/16:0) | 784.6 | 184.1 | 74 | 28 |
| 8.35 | SM(d18:1/22:2) | 783.6 | 184.1 | 50 | 30 |
| 8.36 | PC(16:0/17:1)* | 746.6 | 184.1 | 74 | 28 |
| 8.42 | PE(16:0/22:5) | 766.5 | 625.5 | 18 | 20 |
| 8.48 | PE(18:2/18:1)* | 742.5 | 601.5 | 18 | 20 |
| 8.49 | PC(20:4/17:0) | 796.6 | 184.1 | 74 | 28 |
| 8.52 | PC(18:2/17:0) | 772.6 | 184.1 | 74 | 28 |
| 8.57 | PC(15:1/18:2)* | 742.5 | 184.1 | 74 | 28 |
| 8.57 | PC(16:0/16:0)* | 734.6 | 184.1 | 74 | 28 |
| 8.57 | PC(O-16:0/18:3) | 742.6 | 184.1 | 74 | 28 |
| 8.57 | SM(d18:1/19:1)* | 743.6 | 184.1 | 50 | 30 |
| 8.58 | PC(O-16:0/20:4) | 768.6 | 184.1 | 74 | 28 |
| 8.62 | PC(22:6/18:0) | 834.6 | 184.1 | 74 | 28 |
| 8.63 | PC(O-16:0/18:2) | 744.6 | 184.1 | 74 | 28 |
| 8.63 | SM(d18:2/17:1)* | 713.6 | 184.1 | 50 | 30 |
| 8.66 | SM(d18:1/18:0)* | 731.6 | 184.1 | 50 | 30 |
| 8.68 | PC(20:1/22:6)* | 860.6 | 184.1 | 74 | 28 |
| 8.69 | PC(17:1/20:4) | 794.6 | 184.1 | 74 | 28 |
| 8.7 | PE(17:0/18:2) | 730.5 | 589.5 | 18 | 20 |
| 8.72 | PC(18:2/19:1)* | 798.6 | 184.1 | 74 | 28 |
| 8.74 | PC(16:0/18:1) | 760.6 | 184.1 | 74 | 28 |
| 8.75 | Cer(d18:1/16:0)* | 538.5 | 264.3 | 26 | 32 |
| 8.79 | PE(18:0/22:6)* | 792.6 | 651.5 | 18 | 20 |
| 8.8 | PE(18:0/22:5) | 794.6 | 653.6 | 18 | 20 |
| 8.82 | PC(18:0/22:5) | 836.6 | 184.1 | 74 | 28 |
| 8.84 | SM(d18:2/20:0) | 757.6 | 184.1 | 50 | 30 |
| 8.86 | PC(20:4/18:0) | 810.6 | 184.1 | 74 | 28 |
| 8.86 | SM(d20:0/22:4) | 809.7 | 184.1 | 50 | 30 |
| 8.9 | PC(18:0/18:2) | 786.6 | 184.1 | 74 | 28 |
| 8.93 | PE(16:0/18:1) | 718.5 | 577.5 | 18 | 20 |
| 9.02 | PC(O-16:0/16:1) | 718.6 | 184.1 | 74 | 28 |
| 9.04 | PC(20:0/22:6)* | 862.6 | 184.1 | 74 | 28 |
| 9.04 | PE(20:4/18:0)* | 768.6 | 627.5 | 18 | 20 |
| 9.05 | SM(d16:0/19:0) | 719.6 | 184.1 | 50 | 30 |
| 9.06 | PC(O-16:0/16:0) | 720.6 | 184.1 | 74 | 28 |
| 9.09 | PE(18:2/18:0) | 744.6 | 603.5 | 18 | 20 |
| 9.1 | PC(18:0/20:3) | 812.6 | 184.1 | 74 | 28 |
| 9.11 | Cer(d18:0/14:0) | 512.5 | 266.3 | 26 | 32 |
| 9.11 | PC(18:1/17:0) | 774.6 | 184.1 | 74 | 28 |
| 9.14 | Cer(d18:1/17:0)# | 552.5 | 264.3 | 26 | 32 |
| 9.17 | PC(O-16:0/18:1) | 746.6 | 184.1 | 74 | 28 |
| 9.17 | SM(d18:1/19:0)* | 745.6 | 184.1 | 50 | 30 |
| 9.23 | PC(20:4/19:0) | 824.6 | 184.1 | 74 | 28 |
| 9.25 | PE(18:0/20:3) | 770.6 | 629.6 | 18 | 20 |
| 9.28 | PC(18:2/19:0)* | 800.6 | 184.1 | 74 | 28 |
| 9.29 | PE(17:0/22:6) | 778.5 | 637.5 | 18 | 20 |
| 9.3 | PC(18:0/16:0) | 762.6 | 184.1 | 74 | 28 |
| 9.32 | PC(18:0/22:4)* | 838.6 | 184.1 | 74 | 28 |
| 9.33 | PC(O-18:0/20:4) | 796.6 | 184.1 | 74 | 28 |
| 9.37 | PC(19:1/20:4) | 822.6 | 184.1 | 74 | 28 |
| 9.44 | PC(19:0/22:6) | 848.6 | 184.1 | 74 | 28 |
| 9.44 | SM(d18:1/20:0) | 759.6 | 184.1 | 50 | 30 |
| 9.46 | PC(18:0/18:1)* | 788.6 | 184.1 | 74 | 28 |
| 9.46 | SM(d16:1/24:1)* | 785.7 | 184.1 | 50 | 30 |
| 9.49 | PC(18:0/20:2) | 814.6 | 184.1 | 74 | 28 |
| 9.49 | PE(20:0/20:4) | 796.6 | 655.6 | 18 | 20 |
| 9.5 | Cer(d18:1/18:0)* | 566.6 | 264.3 | 26 | 32 |
| 9.54 | PC(O-16:0/22:4) | 796.6 | 184.1 | 74 | 28 |
| 9.6 | SM(d18:2/24:1) | 811.7 | 184.1 | 50 | 30 |
| 9.62 | PC(16:2/24:1) | 840.6 | 184.1 | 74 | 28 |
| 9.64 | PE(18:0/18:1)* | 746.6 | 605.6 | 18 | 20 |
| 9.77 | PC(O-18:0/16:0) | 748.6 | 184.1 | 74 | 28 |
| 9.8 | PC(20:0/22:4)* | 866.7 | 184.1 | 74 | 28 |
| 9.87 | SM(d19:1/20:0)* | 773.7 | 184.1 | 50 | 30 |
| 9.98 | PC(18:0/18:0) | 790.6 | 184.1 | 74 | 28 |
| 9.99 | SM(d18:2/23:0) | 799.7 | 184.1 | 50 | 30 |
| 10.17 | SM(d18:1/22:0) | 787.7 | 184.1 | 50 | 30 |
| 10.18 | Cer(d18:0/16:0)* | 540.5 | 266.3 | 26 | 32 |
| 10.22 | Cer(d18:1/20:0)* | 594.6 | 264.3 | 26 | 32 |
| 10.26 | PC(16:2/26:1) | 868.7 | 184.1 | 74 | 28 |
| 10.31 | PC(18:1/22:1) | 842.7 | 184.1 | 74 | 28 |
| 10.34 | SM(d18:1/24:1)* | 813.7 | 184.1 | 50 | 30 |
| 10.35 | Cer(d18:2/24:1)* | 646.6 | 262.2 | 26 | 32 |
| 10.35 | PC(20:0/18:1) | 816.6 | 184.1 | 74 | 28 |
| 10.36 | Cer(d18:2/22:0)* | 620.6 | 262.2 | 26 | 32 |
| 10.36 | SM(d17:1/26:1) | 827.7 | 184.1 | 50 | 30 |
| 10.51 | PC(16:2/22:6) | 802.5 | 184.1 | 74 | 28 |
| 10.51 | SM(d17:1/24:0) | 801.7 | 184.1 | 50 | 30 |
| 10.64 | PC(20:5/23:0) | 878.7 | 184.1 | 74 | 28 |
| 10.68 | Cer(d16:1/24:1)* | 620.6 | 236.2 | 26 | 32 |
| 10.71 | Cer(d18:2/23:0) | 634.6 | 262.2 | 26 | 32 |
| 10.72 | PC(19:1/20:1) | 828.6 | 184.1 | 74 | 28 |
| 10.74 | SM(d18:1/26:1) | 841.7 | 184.1 | 50 | 30 |
| 10.84 | PC(19:0/19:0)# | 818.6 | 184.1 | 74 | 28 |
| 10.84 | SM(d18:1/24:0) | 815.7 | 184.1 | 50 | 30 |
| 10.85 | Cer(d18:1/24:1)* | 648.6 | 264.3 | 26 | 32 |
| 10.87 | Cer(d18:1/22:0)* | 622.6 | 264.3 | 26 | 32 |
| 10.92 | PC(18:0/24:2)* | 870.7 | 184.1 | 74 | 28 |
| 11.02 | Cer(d18:2/24:0) | 648.6 | 262.2 | 26 | 32 |
| 11.05 | Cer(d18:0/22:0)* | 624.6 | 266.3 | 26 | 32 |
| 11.16 | SM(d19:0/24:1) | 829.7 | 184.1 | 50 | 30 |
| 11.18 | Cer (d18:1/22:2) | 618.6 | 264.3 | 26 | 32 |
| 11.19 | Cer(d18:1/23:0) | 636.6 | 264.3 | 26 | 32 |
| 11.25 | Cer(d18:2/26:0)* | 676.7 | 262.2 | 26 | 32 |
| 11.47 | Cer(d18:1/24:0)* | 650.6 | 264.3 | 26 | 32 |
| 11.75 | Cer(d18:1/25:0) | 664.7 | 264.3 | 26 | 32 |
| 12.02 | Cer(d18:1/26:0)* | 678.7 | 264.3 | 26 | 32 |
| 12.21 | TG(18:4/16:0/18:2)* | 868.6 | 597.4 | 62 | 24 |
| 12.36 | TG(16:0/16:2/18:1)* | 846.6 | 575.4 | 62 | 24 |
| 12.47 | TG(16:0/14:0/18:3)* | 818.7 | 545.5 | 62 | 24 |
| 12.52 | TG(16:0/16:1/18:3) | 844.6 | 545.4 | 62 | 24 |
| 12.58 | TG(16:0/18:2/18:3) | 870.6 | 597.4 | 62 | 24 |
| 12.63 | TG(18:1/18:2/18:3) | 896.6 | 625.4 | 62 | 24 |
| 12.82 | TG(16:1/14:0/18:1) | 820.7 | 549.4 | 62 | 24 |
| 12.9 | TG(15:0/15:0/15:0)# | 782.6 | 523.4 | 62 | 24 |
| 12.91 | TG(16:0/18:2/18:2) | 872.6 | 573.4 | 62 | 24 |
| 12.96 | TG(18:1/18:2/18:2) | 898.6 | 627.4 | 62 | 24 |
| 13.14 | TG(16:0/14:0/18:1) | 822.6 | 549.4 | 62 | 24 |
| 13.18 | TG(16:0/16:1/18:1) | 848.6 | 575.4 | 62 | 24 |
| 13.23 | TG(16:0/16:0/20:3)* | 874.6 | 603.4 | 62 | 24 |
| 13.26 | TG(16:0/18:1/20:3)* | 900.8 | 629.4 | 62 | 24 |
| 13.35 | TG(18:0/14:0/16:0)* | 824.6 | 551.4 | 62 | 24 |
| 13.37 | TG(16:0/16:0/18:1) | 850.6 | 577.4 | 62 | 24 |
| 13.39 | TG(18:0/16:0/18:2)* | 876.6 | 575.4 | 62 | 24 |
| 13.4 | TG(18:0/18:1/18:2) | 902.6 | 629.4 | 62 | 24 |
| 13.46 | TG(18:0/16:0/16:0) | 852.6 | 579.4 | 62 | 24 |
| 13.48 | TG(18:0/18:1/18:1) | 904.8 | 631.4 | 62 | 24 |
| 13.54 | TG(18:0/18:0/18:1) | 906.6 | 605.4 | 62 | 24 |

* identified with represented standards; # internal standards
